# PCA outperforms popular hidden variable inference methods for molecular QTL mapping

**DOI:** 10.1186/s13059-022-02761-4

**Published:** 2022-10-11

**Authors:** Heather J. Zhou, Lei Li, Yumei Li, Wei Li, Jingyi Jessica Li

**Affiliations:** 1 Department of Statistics, University of California, Los Angeles, Los Angeles, CA 90095 USA; 2 Institute of Systems and Physical Biology, Shenzhen Bay Laboratory, Shenzhen, 518055 China; 3 Division of Computational Biomedicine, Department of Biological Chemistry, School of Medicine, University of California, Irvine, Irvine, CA 92697 USA; 4 Department of Human Genetics, University of California, Los Angeles, Los Angeles, CA 90095 USA; 5 Department of Computational Medicine, University of California, Los Angeles, Los Angeles, CA 90095 USA; 6 Department of Biostatistics, University of California, Los Angeles, Los Angeles, CA 90095 USA

## Abstract

**Background:**

Estimating and accounting for hidden variables is widely practiced as an important step in molecular quantitative trait locus (molecular QTL, henceforth “QTL”) analysis for improving the power of QTL identification. However, few benchmark studies have been performed to evaluate the efficacy of the various methods developed for this purpose.

**Results:**

Here we benchmark popular hidden variable inference methods including surrogate variable analysis (SVA), probabilistic estimation of expression residuals (PEER), and hidden covariates with prior (HCP) against principal component analysis (PCA)—a well-established dimension reduction and factor discovery method—via 362 synthetic and 110 real data sets. We show that PCA not only underlies the statistical methodology behind the popular methods but is also orders of magnitude faster, better-performing, and much easier to interpret and use.

**Conclusions:**

To help researchers use PCA in their QTL analysis, we provide an R package PCAForQTL along with a detailed guide, both of which are freely available at https://github.com/heatherjzhou/PCAForQTL. We believe that using PCA rather than SVA, PEER, or HCP will substantially improve and simplify hidden variable inference in QTL mapping as well as increase the transparency and reproducibility of QTL research.

**Supplementary Information:**

The online version contains supplementary material available at 10.1186/s13059-022-02761-4.

## Background (Section 1)

Genome-wide association studies (GWASs) have identified thousands of genetic variants associated with human traits or diseases [1–4]. However, the majority of GWAS variants are located in non-coding regions of the genome, making it challenging to interpret the GWAS associations [5, 6]. In response to this, molecular quantitative trait locus (molecular QTL, henceforth “QTL”) analysis has emerged as an important field in human genetics, interrogating the relationship between genetic variants and intermediate, molecular traits and potentially explaining GWAS findings [7, 8].

Based on the type of molecular phenotype studied, QTL analyses can be categorized into gene expression QTL (eQTL) analyses [9, 10], alternative splicing QTL (sQTL) analyses [10], three prime untranslated region alternative polyadenylation QTL (3′aQTL) analyses [11], and so on [7, 8]. Among these categories, eQTL analyses, which investigate the association between genetic variants and gene expression levels, are the most common. To date, most (single-tissue) QTL studies are carried out using regression-based methods such as Matrix eQTL [12] and FastQTL [13].

In QTL analysis, a major challenge is that measurements of gene expression levels and other molecular phenotypes can be affected by a number of technical or biological variables other than the genetic variants, such as batch, sex, and age. If these variables are known, then they can be directly included in the QTL pipeline as covariates. However, many of these variables may be unknown or unmeasured. Therefore, it has become standard practice to *first* infer the hidden variables and *then* include the inferred variables as covariates or otherwise account for them in the QTL pipeline [9–11, 14–23] (see Section 5.3 for a numerical example). This type of approach has been shown to both improve the power of QTL identification in simulation settings [24] and empirically increase the number of discoveries in QTL studies [9, 10, 16, 21–23].

**Fig. 1:**
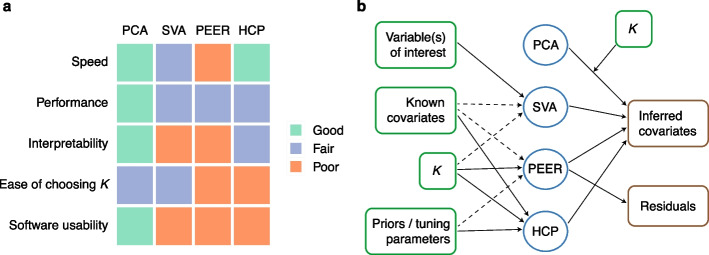
Overall comparison of PCA, SVA, PEER, and HCP and summary of their inputs and outputs. In this work, we use *K* to denote the number of inferred covariates, which are called PCs, SVs, PEER factors, and HCPs in PCA, SVA, PEER, and HCP, respectively. **a** PCA is faster, better-performing, and much easier to interpret and use. For speed and performance comparison, see Section 2.1 (and to a lesser extent, Sections 2.2 and 2.3). For interpretability and ease of choosing *K*, see Sections 2.4 and 2.5, respectively. In terms of software usability, SVA is difficult to apply in QTL settings (Additional file 1: Section S4), PEER is difficult to install, and HCP is poorly documented. In addition, PEER suffers from the disadvantage that there is no consensus in the literature on how it should be used (Additional file 1: Section S4). **b** Inputs (green boxes) and outputs (brown boxes) of the four methods. The fully processed molecular phenotype matrix (after the effects of the known covariates are regressed out in the case of PCA_resid; Table 1) is a required input for all four methods and is thus omitted in the diagram. Dashed arrows indicate optional inputs. PEER outputs both inferred covariates and residuals of the inputted molecular phenotype matrix [32]

Surrogate variable analysis (SVA) [25, 26] is one of the first popular hidden variable inference methods for large-scale genomic analysis. Although initially proposed as a hidden variable inference method for both QTL mapping and differential expression (DE) analysis, currently SVA is primarily used in DE and similar analyses as opposed to QTL mapping [27–30]. We believe this is partly because the SVA package [31] is difficult to apply in QTL settings in that it requires the user to input at least one variable of interest and using too many variables of interest causes the package to fail (Fig. 1; Additional file 1: Section S4); while there are usually at most a few variables of interest in a DE study, there are often millions of single nucleotide polymorphisms (SNPs; variables of interest) in a QTL study. Historically, there have been two versions of the SVA method: two-step SVA [25] and iteratively reweighted SVA (IRW-SVA) [26]; the latter supersedes the former. Therefore, we focus on IRW-SVA in this work.

Probabilistic estimation of expression residuals (PEER) [24, 32] is currently the most popular hidden variable inference method for QTL mapping by far. It is used in the Genotype-Tissue Expression (GTEx) project [9, 10] and many other high-impact studies [11, 14–21]. The PEER method has two main perceived advantages: (1) it can take known covariates into account when estimating the hidden covariates, and (2) its performance does not deteriorate as the number of inferred covariates increases (i.e., it does not “overfit”). One drawback of PEER, though, is that there is no consensus in the literature on *how* it should be used. For example, when there are known covariates available, PEER can be run with or without the known covariates—Stegle et al. [32] do not give an explicit recommendation as to which approach should be used, and both approaches are used in practice (e.g., [9, 10] vs. [11, 16]). Further, PEER outputs both inferred covariates and residuals of the inputted molecular phenotypes (Fig. 1), so the user needs to decide which set of outputs to use (Additional file 1: Section S4; we refer to the approach using the inferred covariates as the “factor approach” and the approach using the residuals as the “residual approach”). Such “flexibility” of PEER could be considered a benefit, but we believe it not only leads to confusion for practitioners who try to use the method but also reduces the transparency and reproducibility of published QTL research.

Hidden covariates with prior (HCP) [33] is another popular hidden variable inference method for QTL mapping. Though less popular than PEER, it has also been used in some high-impact studies [22, 23]. To determine which method is the best and whether PEER indeed has the perceived advantages, we thoroughly evaluate SVA, PEER, and HCP for the first time in the literature. Given that principal component analysis (PCA) [34–38] underlies the methodology behind each of these methods (Section 2.4) and has indeed been used for the same purpose [39, 40], we also include PCA in our evaluation. Through simulation studies (Section 2.1) and real data analysis (Sections 2.2, 2.3 and 2.5), we show that PCA is orders of magnitude faster, better-performing, and much easier to interpret and use (Fig. 1).

## Results (Section 2)

### Comprehensive simulation studies show that PCA is faster and better-performing (Section 2.1)

We compare the runtime and performance of 15 methods (Table 1), including Ideal (assuming the hidden covariates are known), Unadjusted (not estimating or accounting for the hidden covariates), and 13 variants of PCA, SVA, PEER, and HCP, based on two simulation studies. In the first simulation study (Simulation Design 1; Additional file 1: Section S2), we follow the data simulation in Stegle et al. [24]—the original PEER publication—while addressing its data analysis and overall design limitations (Additional file 1: Section S1). In the second simulation study (Simulation Design 2; Additional file 1: Section S3), we further address the data simulation limitations of Stegle et al. [24] (Additional file 1: Section S1) by simulating the data in a more realistic and comprehensive way, roughly following Wang et al. [41]—the SuSiE publication—but introducing the existence of known and hidden covariates. A summary of the main differences between the two simulation designs is provided in Additional file 1: Table S1. The key difference is that in Simulation Design 1, the gene expression levels are primarily driven by trans-regulatory effects rather than cis-regulatory effects or covariate effects (Additional file 1: Table S2), inconsistent with the common belief that trans-regulatory effects are generally weaker than cis-regulatory effects. In contrast, in Simulation Design 2, we focus on cis-QTL detection and carefully control the genotype effects and covariate effects in 176 experiments with two replicates per experiment (Additional file 1: Section S3).

**Table 1:** Summary of the 15 methods we compare based on simulation studies, including﻿ Idea﻿l, Unadjusted, and 13 variants of PCA, SVA, PEER, and HCP (Additional file 1: Section S4). Out of the 15 methods, we select a few representative methods (Section 5.2) for detailed comparison in Simulation Design 2, the abbreviations of which are shown in (D). *Y* denotes the gene expression matrix, $Y_\text {resid}$ denotes the residual matrix outputted by PEER, $X_1$ denotes the known covariate matrix, and $X_2$ denotes the hidden covariate matrix. In Line 3, PCA is run on *Y* directly; in Line 4, PCA is run after the effects of $X_1$ are regressed out from *Y* (Additional file 1: Section S4). The addition signs in (C) denote column concatenation. “filtered” means that we filter out the known covariates that are captured well by the inferred covariates (unadjusted $R^2\ge 0.9$); this filtering is only needed when the hidden variable inference method in (A) does not explicitly take the known covariates into account

	**Inference method**	**Method**	**Response, covariates**	**Method abbr. (if selected)**
	(A)	(B)	(C)	(D)
1		**Ideal**	*Y*, $X_1$ + $X_2$	Ideal
2		**Unadjusted**	*Y*, $X_1$	Unadjusted
3	PCA_direct	**PCA**_direct_screeK	*Y*, $X_1$ (filtered) + top PCs	PCA
4	PCA_resid	**PCA**_resid_screeK	*Y*, $X_1$ + top PCs	
5	SVA_trueK	**SVA**_trueK	*Y*, $X_1$ + SVs	
6	SVA_BE	**SVA**_BE	*Y*, $X_1$ + SVs	SVA
7	PEER_noCov_trueK	**PEER**_noCov_trueK_factors	*Y*, $X_1$ (filtered) + PEER factors	
8	PEER_noCov_trueK	**PEER**_noCov_trueK_residuals	$Y_\text {resid}\,$, NULL	
9	PEER_noCov_largeK	**PEER**_noCov_largeK_factors	*Y*, $X_1$ (filtered) + PEER factors	
10	PEER_noCov_largeK	**PEER**_noCov_largeK_residuals	$Y_\text {resid}\,$, NULL	
11	PEER_withCov_trueK	**PEER**_withCov_trueK_factors	*Y*, $X_1$ + PEER factors	PEER, true K, factors
12	PEER_withCov_trueK	**PEER**_withCov_trueK_residuals	$Y_\text {resid}\,$, NULL	
13	PEER_withCov_largeK	**PEER**_withCov_largeK_factors	*Y*, $X_1$ + PEER factors	
14	PEER_withCov_largeK	**PEER**_withCov_largeK_residuals	$Y_\text {resid}\,$, NULL	PEER, large K, residuals
15	HCP_trueK	**HCP**_trueK	*Y*, $X_1$ + HCPs	HCP

The details of the 15 methods are described in Additional file 1: Section S4, and the evaluation metrics are described in Section 5.1. For convenience, we refer to the simulated molecular phenotypes as gene expression levels throughout our simulation studies; however, they can be interpreted as any type of molecular phenotype after data preprocessing and transformation, e.g., alternative splicing phenotypes and alternative polyadenylation phenotypes (Additional file 1: Table S3).

**Fig. 2:**
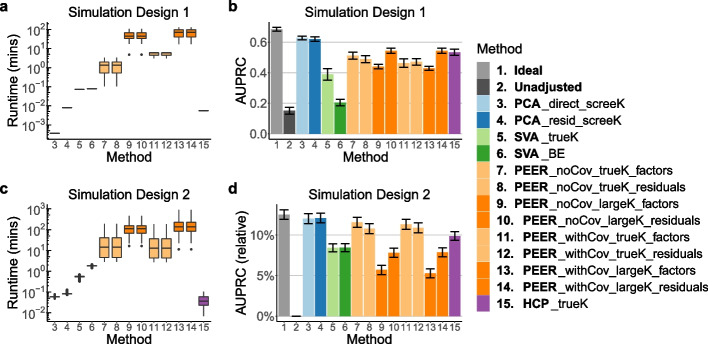
Runtime and AUPRC comparison of all 15 methods (Table 1) in Simulation Design 1 and Simulation Design 2. **a**, **c** PCA and HCP each takes within a few seconds, SVA takes up to a few minutes, and PEER takes up to about 1000 min, equivalent to about 17 h. In particular, PEER takes longer to run when *K* is larger (dark orange vs. light orange boxes). **b**, **d** PCA outperforms SVA, PEER, and HCP in terms of AUPRC. The height of each bar represents the average across simulated data sets. For ease of visualization, in **d**, the *y*-axis displays $\left( \text {AUPRC}-\text {AUPRC}_\text {Unadjusted}\right) /\text {AUPRC}_\text {Unadjusted}$. In this work, error bars indicate standard errors unless otherwise specified (whiskers in box plots are not considered error bars)

**Fig. 3:**
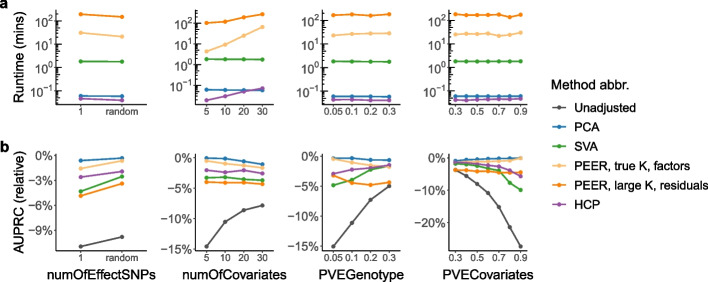
Detailed runtime and AUPRC comparison of the selected representative methods (Table 1) in Simulation Design 2. Each point represents the average across simulated data sets. The *x*-axes are: number of effect SNPs per gene (numOfEffectSNPs), number of simulated covariates (numOfCovariates; including known and hidden covariates), proportion of variance explained by genotype (PVEGenotype), and proportion of variance explained by covariates (PVECovariates) (Additional file 1: Section S3). **a** PCA and HCP are orders of magnitude faster than SVA, which in turn is orders of magnitude faster than PEER. **b** PCA outperforms SVA, PEER, and HCP in terms of AUPRC across different simulation settings. For ease of visualization, the *y*-axis displays $\left( \text {AUPRC}-\text {AUPRC}_\text {Ideal}\right) /\text {AUPRC}_\text {Ideal}$. Consistent with our expectation, the performance gap between Unadjusted and Ideal is the largest (and thus accounting for hidden covariates is the most important) when numOfCovariates is small, when PVEGenotype is small, and when PVECovariates is large

The results from our simulation studies are summarized in Figs. 2 and 3 and Additional file 1: Figs. S1, S3, and S4. We find that PCA and HCP are orders of magnitude faster than SVA, which in turn is orders of magnitude faster than PEER, and that PCA outperforms SVA, PEER, and HCP in terms of the area under the precision-recall curve (AUPRC) of the QTL result (Figs. 2 and 3). On a dataset-by-dataset basis, PCA outperforms the other methods in terms of AUPRC in 11% to 88% of the simulated data sets and underperforms them in close to 0% of the simulated data sets in Simulation Design 2 (Additional file 1: Fig. S3d). In addition, PCA has the highest average concordance scores, a metric for the concordance between the true hidden covariates and the inferred covariates (Section 5.1; Additional file 1: Figs. S1 and S4), which explains why PCA performs the best in terms of AUPRC.

To contrast the results in Stegle et al. [24], we also compare the powers of the different methods in Simulation Design 1 (Additional file 1: Fig. S1). We find that PCA is more powerful than SVA, PEER, and HCP. Notably, SVA and PEER have very low power in identifying trans-QTL relations—an especially unfavorable result for SVA and PEER, considering that the gene expression levels are primarily driven by trans-regulatory effects in Simulation Design 1 (Additional file 1: Table S2).

Incidentally, Fig. 2 and Additional file 1: Fig. S3 also provide us with the following insights into the different ways of using PEER (Additional file 1: Section S4). First, running PEER *with* the known covariates has no advantage over running PEER *without* the known covariates in terms of AUPRC, given the choice of *K* (the number of inferred covariates) and the choice between the factor approach and the residual approach. In fact, running PEER *with* the known covariates significantly increases the runtime of PEER in real data (Section 2.3). Second, contrary to claims in Stegle et al. [24, 32], the performance of PEER *does* deteriorate as the number of PEER factors increases. The only exception is when the residual approach is used in Simulation Design 1 (Fig. 2). But given that Simulation Design 2 is more realistic than Simulation Design 1 and that the factor approach is more popular than the residual approach [9–11, 17–20], the take-home message should be that in general, the performance of PEER is worse when we use a large *K* rather than the true *K*. Third, whether the factor approach or the residual approach performs better depends on the choice of *K*. When we use the true *K*, the factor approach performs better, but when we use a large *K*, the residual approach performs better. All in all, PCA outperforms all different ways of using PEER in both of our simulation studies (Fig. 2).

### PEER factors sometimes fail to capture important variance components of the molecular phenotype data (Section 2.2)

For our real data analysis, we examine the most recent GTEx eQTL and sQTL data [10] (Sections 2.3 and 2.5) and the 3′aQTL data prepared by Li et al. [11] from GTEx RNA-seq reads [9] (Section 2.2). While the exact data analysis pipelines are different (Additional file 1: Table S3), these studies all choose PEER as their hidden variable inference method.

Unlike PCs, which are always uncorrelated (Additional file 1: Section S5.1), PEER factors are not guaranteed to be uncorrelated. Here we show through the above-mentioned 3′aQTL data that PEER factors can be highly correlated with each other (to the extent that many or all of them are practically identical) and thus fail to capture important variance components of the molecular phenotype data.

Given a post-imputation alternative polyadenylation phenotype matrix (each entry is between zero and one, representing a proportion), Li et al. [11] run PEER without further data transformation using the number of PEER factors chosen by GTEx [9] (Additional file 1: Table S3). To assess the impact of data transformation on the PEER factors, we also run PEER after transforming the data in three ways: (1) center and scale (to unit variance) each feature, (2) apply inverse normal transform (INT) [42] to each feature (“INT within feature”), and (3) apply INT to each sample (“INT within sample”). Among these methods, GTEx [9, 10] uses “INT within feature” for its eQTL data and “INT within sample” for its sQTL data (Additional file 1: Table S3). To quantify how many “distinct” or “nonrepetitive” PEER factors there are, given a set of PEER factors, we group them into clusters such that in each cluster, the correlation between any two PEER factors is above a pre-defined threshold (0.99, 0.9, or 0.8) in absolute value (this is done via hierarchical clustering [43] with complete linkage and the distance defined as one minus the absolute value of the correlation). Therefore, the number of PEER factor clusters can be interpreted as the number of distinct or nonrepetitive PEER factors.

**Fig. 4:**
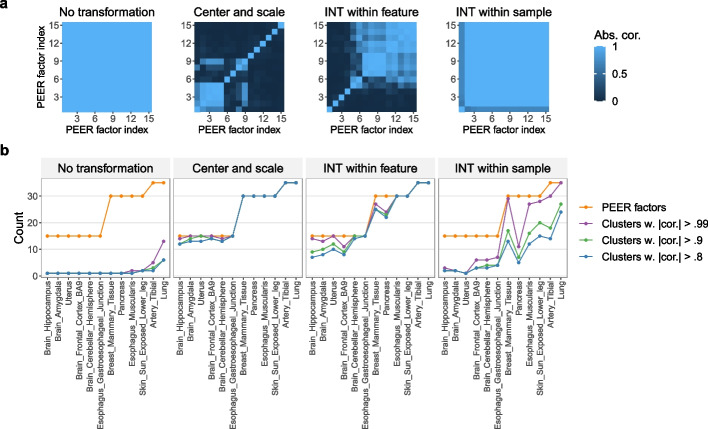
In the 3′aQTL data prepared by Li et al. [11] from GTEx RNA-seq reads [9], PEER factors can be highly correlated with each other to the extent that many or all of them are practically identical. **a** Correlation heatmaps of PEER factors for Brain_Hippocampus. For ease of visualization, the PEER factors are reordered based on results from hierarchical clustering (Section 2.2) in each heatmap. **b** The *x*-axis shows 12 randomly selected tissue types with increasing sample sizes. The *y*-axis shows the number of PEER factors requested (orange line) or the number of PEER factor clusters. Given a set of PEER factors, we group them into clusters such that in each cluster, the correlation between any two PEER factors is above 0.99, 0.9, or 0.8 in absolute value (Section 2.2). Therefore, the number of PEER factor clusters can be interpreted as the number of distinct or nonrepetitive PEER factors. We find that in many cases, the number of distinct PEER factors is considerably smaller than the number of PEER factors requested, and when this issue is severe (e.g., “No transformation” and “INT within sample”), the PEER factors fail to capture important variance components of the molecular phenotype data (Additional file 1: Fig. S5)

Our results show that in many cases, the number of distinct PEER factors is considerably smaller than the number of PEER factors requested (Fig. 4), and when this issue is severe (e.g., “No transformation” and “INT within sample”), the PEER factors fail to capture important variance components of the molecular phenotype data (Additional file 1: Fig. S5). Since the numbers of discoveries increase substantially with the numbers of PEER factors in GTEx’s eQTL analyses [9, 10], where the PEER factors are essentially identical to PCs (Section 2.3), it is possible that replacing the nearly-all-identical PEER factors with appropriate numbers of PCs in Li et al. [11]’s 3′aQTL analysis can lead to more discoveries. This is a potential direction for a future study.

### PEER factors are almost identical to PCs but take three orders of magnitude longer to compute in GTEx eQTL and sQTL data (Section 2.3)

**Fig. 5:**
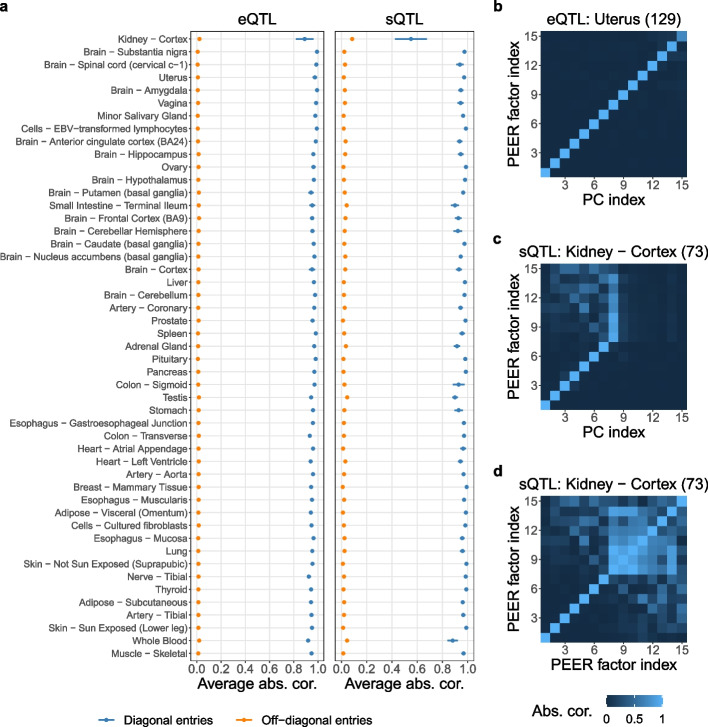
PEER factors are almost identical to PCs in GTEx eQTL and sQTL data [10]. **a** The *y*-axis shows all 49 tissue types with GTEx QTL analyses ordered by sample size (from small to large). Given a fully processed molecular phenotype matrix, we summarize the correlation matrix (in absolute value) between the PEER factors obtained and used by GTEx and the top PCs into two numbers: the average of the diagonal entries and the average of the off-diagonal entries. With the exception of Kidney - Cortex sQTL data, the diagonal entries have averages close to one, and the off-diagonal entries have averages close to zero (both have minimal standard errors). **b** A typical correlation heatmap showing near-perfect one-to-one correspondence between the PEER factors and the top PCs. **c** In Kidney - Cortex sQTL data, the PEER factors and the top PCs do not have a perfect one-to-one correspondence. The reason is because the PEER factors are highly correlated with each other (**d**), while PCs are always uncorrelated (Additional file 1: Section S5.1). The numbers in parentheses represent sample sizes. To produce this figure, we reorder the PEER factors based on the PCs (Additional file 1: Algorithm S1), although in almost all cases, this reordering does not change the original ordering of the PEER factors because PEER initializes with PCs [24]

We report the surprising finding that in both GTEx eQTL and sQTL data [10], the PEER factors obtained by GTEx and used in its QTL analyses are almost identical to PCs. Specifically, given a fully processed molecular phenotype matrix, there is almost always a near-perfect one-to-one correspondence between the PEER factors and the top PCs (Fig. 5). This means that after the variational Bayesian inference in PEER initializes with PCs [24], it does not update the PCs much beyond scaling them (see Section 2.4 for an explanation). Therefore, it is no surprise that replacing the PEER factors with PCs in GTEx’s FastQTL pipeline [10, 13] does not change the QTL results much (Additional file 1: Figs. S6 and S7) because in linear regressions (the basis of both Matrix eQTL [12] and FastQTL [13]), scaling and/or shifting the predictors does not change the *p*-values of *t*-tests for non-intercept terms (neither does scaling and/or shifting the response, for that matter).

However, PEER is at least three orders of magnitude slower than PCA (Additional file 1: Fig. S6). For a given expression matrix, running PEER without the known covariates (GTEx’s approach) takes up to about 32 hours, while running PCA (with centering and scaling; our approach) takes no more than a minute.

To draw a connection between our simulation results and real data results, we analyze them jointly in Additional file 1: Fig. S8 and make the following two key observations. First, we find that in the simulation studies, PCA almost always outperforms PEER in terms of AUPRC (confirming our results in Section 2.1), and the percentage of QTL discoveries shared between PEER and PCA is a good predictor of the relative performance of PEER versus PCA—the higher the percentage of QTL discoveries shared, the smaller the performance gap between PEER and PCA. Second, the percentages of QTL discoveries shared between the two methods in GTEx eQTL data [10] fall comfortably within the range of percentage of QTL discoveries shared in Simulation Design 2. These two observations together suggest that PCA likely outperforms PEER in GTEx eQTL data [10] even though the results largely overlap.

### PCA, SVA, PEER, and HCP are closely related statistical methods (Section 2.4)

**Fig. 6:**
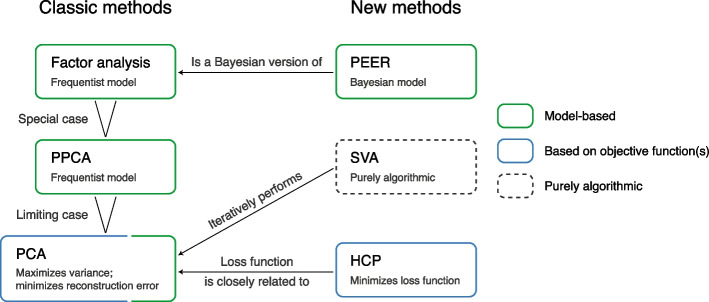
PCA, SVA, PEER, and HCP are closely related statistical methods despite their apparent dissimilarities. In particular, the methodology behind SVA, PEER, and HCP can all be traced back to PCA. PCA [34–38] is traditionally derived by optimizing some objective functions (either maximum variance or minimum reconstruction error; Additional file 1: Section S5.1), but more recently, it is shown that PCA can be derived as a limiting case of probabilistic principal component analysis (PPCA) [45], which in turn is a special case of factor analysis [35, 46]. PEER [24, 32] is based on a Bayesian probabilistic model and can be considered a Bayesian version of factor analysis. SVA [25, 26] is purely algorithmic and is not defined based on a probabilistic model or objective function. The steps of the SVA algorithm are complicated [44], but in a nutshell, SVA iterates between two steps: (1) reweight the features of the molecular phenotype matrix, and (2) perform PCA on the resulting matrix (with centering but without scaling) [26]. Lastly, HCP [33] is defined by minimizing a loss function that is very similar to the minimum-reconstruction-error loss function of PCA (Additional file 1: Section S5.2)

We report that PCA, SVA, PEER, and HCP are closely related statistical methods despite their apparent dissimilarities. In particular, the methodology behind SVA, PEER, and HCP can all be traced back to PCA (Fig. 6). We have previously reviewed these methods in detail in Zhou [44]. Here we aim to provide a brief summary and highlight their connections.

PCA [34–38] is traditionally derived by optimizing some objective functions (either maximum variance or minimum reconstruction error; Additional file 1: Section S5.1), but more recently, it is shown that PCA can be derived as a limiting case of probabilistic principal component analysis (PPCA) [45], which in turn is a special case of factor analysis [35, 46]—a dimension reduction method commonly used in psychology and the social sciences that is based on a *frequentist* probabilistic model.

PEER [24, 32] is based on a *Bayesian* probabilistic model and can be considered a Bayesian version of factor analysis (with the not-very-useful ability to explicitly model the known covariates; see Section 2.1 for why we do not find this ability useful). Inference is performed using variational Bayes and initialized with the PCA solution [24]. Given that PCA underlies the PEER model (Fig. 6) and PEER initializes with PCs, it is not surprising that PEER factors are almost identical to PCs in GTEx eQTL and sQTL data [10] (Section 2.3).

SVA [25, 26] is purely algorithmic and is not defined based on a probabilistic model or objective function. The steps of the SVA algorithm are complicated [44], but in a nutshell, SVA iterates between two steps: (1) reweight the features of the molecular phenotype matrix, and (2) perform PCA on the resulting matrix (with centering but without scaling) [26].

Lastly, HCP [33] is defined by minimizing a loss function that is very similar to the minimum-reconstruction-error loss function of PCA (Additional file 1: Section S5.2). The optimization is done through coordinate descent with one deterministic initialization (see source code of the HCP R package [33]). In short, SVA, PEER, and HCP can all be considered extensions or more complex versions of PCA, though we show that the complexity is a burden rather than a benefit (Fig. 1).

### PCA provides insight into the choice of *K *(Section 2.5)

Choosing *K*, the number of inferred covariates in the context of hidden variable inference or the number of dimensions or clusters in more general contexts, is always a difficult task. Nonetheless, based on the proportion of variance explained (PVE) by each PC (Additional file 1: Section S5.1), PCA offers convenient ways of choosing *K* such as the elbow method and the Buja and Eyuboglu (BE) algorithm [47] (more details below). Since SVA is heavily based on PCA (Section 2.4), it is able to adapt and make use of the BE algorithm. In contrast, PEER and HCP do not offer easy ways of choosing *K*; for lack of a better method, users of PEER and HCP often choose *K* by maximizing the number of discoveries [9, 10, 16, 21–23]. Not only is this approach of choosing *K* extremely computationally expensive and theoretically questionable, here we also show from the perspective of PCA that it may yield inappropriate choices of *K*.

**Fig. 7:**
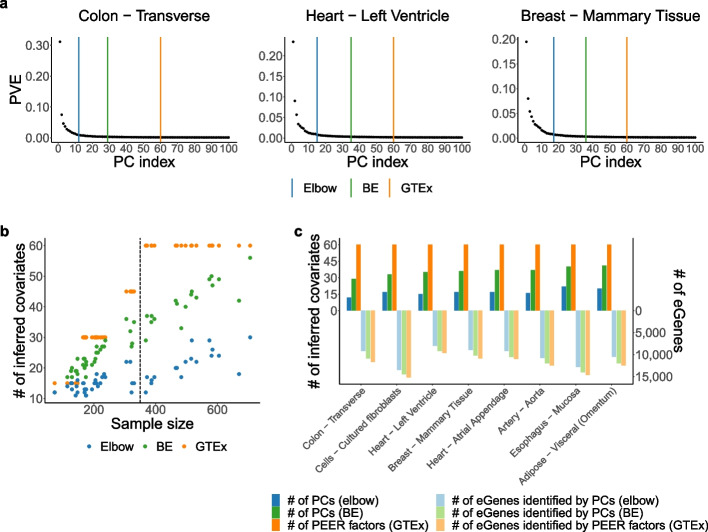
PCA provides insight into the choice of *K*. Recall from Section 2.3 that PEER factors are almost identical to PCs in GTEx eQTL data [10]. Therefore, for each tissue type, we compare the number of PEER factors selected by GTEx to (1) the number of PCs chosen via an automatic elbow detection method (Additional file 1: Algorithm S2) and (2) the number of PCs chosen via the BE algorithm (Additional file 1: Algorithm S3; the default parameters are used). **a** Example scree plots. **b** This scatter plot contains 49 dots of each color, corresponding to the 49 tissue types with GTEx eQTL analyses. The number of PEER factors selected by GTEx far exceeds the number of PCs chosen via BE for many tissue types with sample size above 350 (dashed line), suggesting that the number of PEER factors selected by GTEx may be too large. **c** For the eight tissue types with the largest absolute differences between the number of PEER factors chosen by GTEx and the number of PCs chosen via BE (all eight tissue types have sample size above 350), we replace the PEER factors with smaller numbers of PCs in GTEx’s FastQTL pipeline [10, 13] and find that we can reduce the number of inferred covariates to between 20% ($12/60=20\%$, Colon - Transverse) and 40% ($22/60\approx 36.67\%$, Esophagus - Mucosa) of the number of PEER factors selected by GTEx without significantly reducing the number of discovered cis-eGenes

Recall from Section 2.3 that PEER factors are almost identical to PCs in GTEx eQTL data [10] (the number of PEER factors is chosen by maximizing the number of discovered cis-eGenes for each pre-defined sample size bin; Additional file 1: Table S3). Therefore, for each tissue type, we compare the number of PEER factors selected by GTEx to (1) the number of PCs chosen via an automatic elbow detection method (Additional file 1: Algorithm S2) and (2) the number of PCs chosen via the BE algorithm (Additional file 1: Algorithm S3; the default parameters are used). The BE algorithm is a permutation-based approach for choosing *K* in PCA. Intuitively, it retains PCs that explain more variance in the data than by random chance and discards those that do not. Hence, based on the statistical interpretation of the BE algorithm and the scree plots (examples shown in Fig. 7), we believe that the number of PCs chosen via BE should be considered an upper bound of the reasonable number of PCs to choose in GTEx eQTL data [10].

Our results show that the number of PEER factors selected by GTEx is almost always greater than the number of PCs chosen via *BE*, which in turn is almost always greater than the number of PCs chosen via *elbow* (Fig. 7). In particular, the number of PEER factors selected by GTEx far exceeds the number of PCs chosen via *BE* for many tissue types with sample size above 350, suggesting that the number of PEER factors selected by GTEx may be too large. This hypothesis is further supported by the fact that we can reduce the number of inferred covariates to between 20% and 40% of the number of PEER factors selected by GTEx without significantly reducing the number of discovered cis-eGenes (Fig. 7).

## Discussion (Section 3)

Hidden variable inference is widely practiced as an important step in QTL mapping for improving the power of QTL identification. Popular hidden variable inference methods include SVA, PEER, and HCP. In this work, we show that PCA not only underlies the statistical methodology behind the popular methods (Section 2.4) but is also orders of magnitude faster, better-performing, and much easier to interpret and use (Fig. 1; relatedly, Malik and Michoel [48] have pointed out issues with the optimization algorithm used in PANAMA [49]—a variant of PEER, and the computational efficiency of PCA has been reported in other settings, including genomic selection [50]). Our conclusions are consistent with those from Cuomo et al. [51], who conclude that PCA is superior to alternative hidden variable inference methods for improving the power of single-cell eQTL analysis.

On the simulation front, we compare the runtime and performance of PCA, SVA, PEER, and HCP via two simulation studies (Section 2.1). In the first simulation study, we follow the data simulation in Stegle et al. [24], the original PEER publication, while addressing its data analysis and overall design limitations. In the second simulation study, we further address the data simulation limitations of Stegle et al. [24] by simulating the data in a more realistic and comprehensive way. Both simulation studies unanimously show that PCA is faster and better-performing. Further, they show that running PEER *with* the known covariates has no advantage over running PEER *without* the known covariates—in fact, running PEER *with* the known covariates makes PEER significantly slower (Additional file 1: Fig. S6)—and that contrary to claims in Stegle et al. [24, 32], the performance of PEER *does* deteriorate as the number of PEER factors increases (Section 2.1). One caveat of our simulation studies, though, is that the genotype and covariates all have linear effects on the gene expression levels (consistent with Stegle et al. [24] and Wang et al. [41]). But since PCA, SVA, PEER, and HCP are all linear methods or assume linearity [44], and so does linear regression, we do not believe our conclusions would change qualitatively if we simulated the data in a nonlinear fashion.

On the real data front, we examine the most recent GTEx eQTL and sQTL data [10] and the 3′aQTL data prepared by Li et al. [11] from GTEx RNA-seq reads [9]. While the exact data analysis pipelines are different (Additional file 1: Table S3), these studies all choose PEER as their hidden variable inference method (due to lack of data availability, we do not examine more real data sets). Our analysis shows that PEER, the most popular hidden variable inference method for QTL mapping currently, produces nearly identical results as PCA at best (Section 2.3), is at least three orders of magnitude slower than PCA (Additional file 1: Fig. S6), and can be full of pitfalls. Specifically, we show that in certain cases, PEER factors can be highly correlated with each other and thus fail to capture important variance components of the molecular phenotype data, leading to potential loss of power in QTL identification (Section 2.2). Further, we show from the perspective of PCA that choosing the number of PEER factors by maximizing the number of discoveries (a common approach used by practitioners) may yield inappropriate choices of *K*, leading to model overfit and potential loss of power and precision (Section 2.5).

Between the two PCA approaches, PCA_direct (running PCA on the fully processed molecular phenotype matrix *directly* and filtering out the known covariates that are captured well by the top PCs afterwards) and PCA_resid (running PCA after regressing out the effects of the known covariates from the molecular phenotype matrix) (Table 1; Additional file 1: Section S4), we recommend PCA_direct because the two approaches perform similarly in our simulation studies and PCA_direct is simpler. In addition, PCA_direct can better hedge against the possibility that the known covariates are not actually important confounders because in PCA_direct, the known covariates do not affect the calculation of the PCs. We also advise the users to make sure to center and scale their data when running PCA unless they are experts and have a good reason not to.

In addition to the benefits discussed so far, using PCA rather than SVA, PEER, or HCP has another conceptual benefit. While SVA, PEER, and HCP are hidden variable inference (i.e., factor discovery) methods, PCA can be interpreted and used as both a *dimension reduction* and a *factor discovery* method. Therefore, PCs of the molecular phenotype data need not be considered inferred covariates; instead, they can be considered a dimension-reduced version of the molecular phenotype data—by including them as covariates, we are controlling for the effect of the overall gene expression profile on the expression level of any individual gene (taking expression phenotypes as an example). With this perspective, including *phenotype* PCs as covariates is analogous to including *genotype* PCs as covariates (which is commonly done to correct for population stratification [9, 10]). This perspective solves the conundrum that inferred covariates such as PEER factors are often difficult to interpret using known technical and biological variables [52].

## Conclusions (Section 4)

To help researchers use PCA in their QTL analysis, we provide an R package PCAForQTL, which implements highly interpretable methods for choosing the number of PCs (Additional file 1: Algorithms S2 and S3), a graphing function, and more, along with a detailed tutorial. Both resources are freely available at https://github.com/heatherjzhou/PCAForQTL [53]. We believe that using PCA rather than SVA, PEER, or HCP will substantially improve and simplify hidden variable inference in QTL mapping as well as increase the transparency and reproducibility of QTL research.

## Methods (Section 5)

### Evaluation metrics (Section 5.1)

Given a simulated data set, we evaluate each of the 15 methods in Table 1 mainly in three ways (when applicable): runtime, AUPRC, and adjusted $R^2$ measures (including adjusted $R^2$, reverse adjusted $R^2$, and concordance score).

First, we record the runtime of the hidden variable inference step (Additional file 1: Section S4; not applicable for Ideal and Unadjusted).

Second, we calculate the area under the precision-recall curve (AUPRC) of the QTL result. We use AUPRC rather than the area under the receiver operating characteristic curve (AUROC) because AUPRC is more appropriate for data sets with imbalanced classes (there are far more negatives than positives in our simulated data sets and in QTL settings in general). Since AUPRC measures the trade-off between the true positive rate (i.e., power) and the false discovery rate (i.e., one minus precision), it is a more comprehensive metric than power. However, to contrast the results in Stegle et al. [24], we also compare the powers of the different methods in Simulation Design 1.

Third, for each simulated data set, each method except Ideal and Unadjusted gets an adjusted $R^2$ score (short as “adjusted $R^2$”), a reverse adjusted $R^2$ score (short as “reverse adjusted $R^2$”), and a concordance score. The adjusted $R^2$ score summarizes how well the true hidden covariates can be captured by the inferred covariates; the reverse adjusted $R^2$ score summarizes how well the inferred covariates can be captured by the true hidden covariates (a low score indicates that the inferred covariates are invalid or “meaningless”); lastly, the concordance score is the average of the previous two scores and thus measures the concordance between the true hidden covariates and the inferred covariates. Specifically, given *m* true hidden covariates and *n* inferred covariates, first, we calculate *m* adjusted $R^2$’s (regressing each true hidden covariate against the inferred covariates) and *n* reverse adjusted $R^2$’s (regressing each inferred covariate against the true hidden covariates); then, we average the *m* adjusted $R^2$’s to obtain the adjusted $R^2$ score and average the *n* reverse adjusted $R^2$’s to obtain the reverse adjusted $R^2$ score; finally, we define the concordance score as the average of the adjusted $R^2$ score and the reverse adjusted $R^2$ score.

### Selection of representative methods for detailed comparison (Section 5.2)

Here we describe how we select a few representative methods from the 15 methods for detailed comparison in Simulation Design 2 (Table 1). From Fig. 2d and Additional file 1: Fig. S3, we see that the two PCA methods perform almost identically, so for simplicity, we select PCA_direct_screeK. The two SVA methods perform almost identically as well, so we select SVA_BE. For PEER, whether the known covariates are inputted when PEER is run has little effect on the AUPRC. Further, we observe that when we use the true *K*, the factor approach outperforms the residual approach, but when we use a large *K*, the residual approach outperforms the factor approach. Therefore, we select PEER_withCov_trueK_factors and PEER_withCov_largeK_residuals as the representative PEER methods. In addition, Ideal, Unadjusted, and HCP_trueK are selected.

### A numerical example (Section 5.3)

Here we provide a simple numerical example of QTL analysis with hidden variable inference by summarizing the setup of GTEx’s cis-eQTL analysis for Colon - Transverse [10].

Let *Y* denote the $n\times p$ fully processed gene expression matrix with $n=368$ samples and $p=25{,}379$ genes. Let $X_1$ denotes the $n\times K_1$ known covariate matrix with $K_1=8$ known covariates, which include the top five genotype PCs, WGS sequencing platform (HiSeq 2000 or HiSeq X), WGS library construction protocol (PCR-based or PCR-free), and donor sex. Let $X_{\text {inferred}}$ denote the $n\times K$ inferred covariate matrix with $K=60$ PEER factors, which are obtained by running PEER on *Y* (Additional file 1: Table S3). For gene *j*, $j=1,\cdots ,p\,$, the relevant genotype data is stored (conceptually speaking) in $S_j\,$, the $n\times q_j$ genotype matrix, where each column of $S_j$ corresponds to a local common SNP for gene *j*, and $q_j$ is typically under 15,000.

Given these input data, the nominal pass (the first step) of FastQTL [13], or equivalently, Matrix eQTL [12], performs a linear regression for each gene and each of its local common SNPs. Specifically, for $j=1,\cdots ,p\,$, $l=1\cdots ,q_j\,$, the linear regression represented by the following R lm() formula is run:

1 $$\begin{aligned} Y[\,,\,j] \;\sim \; S_{j}[\,,\,l]\,+\,X_{1}\,+\,X_{\text {inferred}} \end{aligned}$$

(where $Y[\,,\,j]$ denotes the *j*th column of *Y*, and $S_j[\,,\,l]$ denotes the *l*th column of $S_j$), and the *p*-value for the null hypothesis that the coefficient corresponding to $S_j[\,,\,l]$ is zero (given the covariates) is retained. The top five genotype PCs in $X_1$ are included in the analysis to correct for population stratification [9, 10] and are typically considered known covariates (see Section 3).

## Supplementary information

**Additional file 1:** Supplementary materials. Includes all supplementary text, figures, tables, and algorithms.

**Additional file 2:** Review history.

## Data Availability

The R package PCAForQTL and a tutorial on using PCA for hidden variable inference in QTL mapping are available at https://github.com/heatherjzhou/PCAForQTL [53]. The code used to generate the results in this work is available at https://doi.org/10.5281/zenodo.6788888 [54]. In addition, this work makes use of the following data and software: $\bullet$ GTEx V8 public data [10], including fully processed gene expression matrices, fully processed alternative splicing phenotype matrices, known covariates, PEER factors, and QTL results, are downloaded from https://gtexportal.org/home/datasets. $\bullet$ GTEx V8 protected data [10], specifically, the whole genome sequencing (WGS) phased genotype data, are downloaded from the AnVIL repository with an approved dbGaP application (see https://gtexportal.org/home/protectedDataAccess). $\bullet$ 3′aQTL data prepared by Li et al. [11] from GTEx RNA-seq reads [9] are available from the authors by request. $\bullet$ SVA R package Version 3.40.0 (https://bioconductor.org/packages/sva/, accessed on October 15, 2021). $\bullet$ PEER R package Version 1.3 (https://bioconda.github.io/recipes/r-peer/README.html, accessed before October 15, 2021). $\bullet$ HCP R package Version 1.6 (https://rdrr.io/github/mvaniterson/Rhcpp/, accessed on October 15, 2021). $\bullet$ FastQTL (https://github.com/francois-a/fastqtl, accessed before October 15, 2021).
