# Supplementary material for: PCA outperforms popular hidden variable inference methods for molecular QTL mapping
Source: Genome Biol. 2022 Oct 11;23:210. doi: 10.1186/s13059-022-02761-4 (PMC9552461; doi:10.1186/s13059-022-02761-4)
Supplement: Supplementary file 1 — Additional file 1: Supplementary materials. Includes all supplementary text, figures, tables, and algorithms. [file 13059_2022_2761_MOESM1_ESM.pdf]

Supplementary materials for

PCA outperforms popular hidden variable inference methods for molecular QTL mapping

Heather J. Zhou, Lei Li, Yumei Li, Wei Li, Jingyi Jessica Li

S1 Limitations of the original PEER simulation study

The simulation study in the original PEER publication [1] is limited. We categorize its limitations into three categories: (1) data analysis limitations, (2) overall design limitations, and (3) data simulation limitations.

The data analysis limitations include:

(a) The study only compares PEER against the other methods in terms of power, not in terms of false positive rate or false discovery rate (see Methods for our evaluation metrics).
(b) The study does not use PCA or SVA properly (we do; Section S4).
(c) The study does not evaluate the different ways of using PEER (we do; Section S4).
(d) The study uses ad hoc priors for PEER that are different from the default priors (we use the default priors; Section S4).

The overall design limitations include:

(a) The study only simulates one replicate of one experiment. That is, the entire simulation study is based on one simulated data set (we simulate 10 replicates in our first simulation study; Section S2).

The data simulation limitations include (see Table S1 for our solutions):

(a) The data dimensions are minimal, with $q = 100$ SNPs in the entire genome.
(b) The SNP genotypes are simulated independently and identically with a target minor allele frequency (MAF) of 0.4, so there is no linkage disequilibrium (LD) and a higher average MAF than in real data (the average MAF in GTEx data [2], after SNPs with MAF under 0.01 are filtered out, is about 0.15; Section S3.1).
(c) The gene expression levels are primarily driven by trans-regulatory effects rather than cis-regulatory effects or covariate effects (Table S2), inconsistent with the common belief that trans-regulatory effects are generally weaker than cis-regulatory effects.

In addition, the simulation study in the original PEER publication [1] is imperfect in that the description of the data simulation and analysis is vague and inconsistent, and there is no reproducible code. In contrast, we describe our data simulation and analysis in detail (Sections S2 to S4) and provide the code we use to generate the results (see Availability of data and materials).

S2 Simulation Design 1

S2.1 Data simulation

In Simulation Design 1, we perform 10 replicates of the same experiment, where in each replicate, we follow the data simulation in Stegle et al. [1] as closely as possible.

In each replicate, we simulate a data set with $n = 80$ individuals, $p = 400$ genes, $q = 100$ SNPs in the entire genome, $K_1 = 3$ known covariates, and $K_2 = 7$ hidden covariates. Let i, j, l, and k be the indices of individuals, genes, SNPs, and covariates, respectively. That is, $i = 1, \cdots, n$; $j = 1, \cdots, p$; $l = 1, \cdots, q$; and $k = 1, \cdots, (K_1 + K_2)$. The data simulation consists of three steps.

In the first step, we simulate $Y_{\text{beforeDSE}}$, the gene expression matrix before downstream effect, based on

$$\underset{n\times p}{Y_{\text{beforeDSE}}} = \underset{n\times q}{S}\left(\underset{q\times p}{I_1} \odot \underset{q\times p}{B_1}\right) + \underset{n\times(K_1+K_2)}{X} \underset{(K_1+K_2)\times p}{B_2} + \underset{n\times p}{E}, \tag{S1}$$

where $\odot$ denotes element-wise multiplication. Specifically, in the genotype component, we have

- S: genotype matrix. Each entry is drawn independently from $Binom(2, \text{prob} = 0.4)$. That is, the target MAF is 0.4. In this work, all random sampling is independent unless otherwise specified.
- I_1: effect indicator matrix. Each entry is drawn from $Ber(0.01)$.
- B_1: effect size matrix. Each entry is drawn from $N(0, \text{var} = 4)$.

In the covariate component, we have

- X: covariate matrix. Each entry is drawn from $N(0, \text{var} = 0.6)$. The first K_1 columns are designated as the known covariates (X_1, $n \times K_1$), and the last K_2 columns are designated as the hidden covariates (X_2, $n \times K_2$).
- B_2: effect size matrix. First, we draw $\sigma_k^2 \sim 0.8\,(\Gamma(\text{shape} = 2.5, \text{rate} = 0.6))^2$, the covariate-specific effect size variance. Then, we draw $(B_2)_{kj} \sim N\left(0, \text{var} = \sigma_k^2\right)$.

Lastly, in the noise component, we have

- E: noise matrix. First, we draw $\tau_j \sim \Gamma(\text{shape} = 3, \text{rate} = 1)$, the gene-specific noise precision. Then, we draw $(E)_{ij} \sim N\left(0, \text{var} = 1/\tau_j\right)$.

In the second step, we simulate Y_{DSE}, the gene expression matrix due to the downstream effect of

genes, based on

$$\underset{n\times p}{Y_{\text{DSE}}} = \underset{n\times p}{Y_{\text{beforeDSE}}} \left(\underset{p\times p}{I_3} \odot \underset{p\times p}{B_3} \right), \tag{S2}$$

where we have

- I_3: effect indicator matrix. To simulate I_3, we start with a zero matrix. Then, we randomly choose three rows corresponding to genes with at least one cis-QTL (Section S2.2). For each of these three rows, we randomly assign 30 entries corresponding to genes other than the current gene in consideration (avoiding self-loops) to be one.
- B_3: effect size matrix. Each entry is drawn from $N(8, \text{var} = 0.8)$ for "strong downstream effects" [1].

As we see in Section S2.2, the downstream effect of genes induces trans-QTL relations.

In the third and last step, we define Y, the final, observed gene expression matrix, as

$$\underset{n\times p}{Y} = \underset{n\times p}{Y_{\text{beforeDSE}}} + \underset{n\times p}{Y_{\text{DSE}}}. \tag{S3}$$

S2.2 Definition of truth

In a simulated data set, the cis-QTL relations are encoded in the $q \times p$ binary matrix I_1. The lj-th entry being one means that SNP l and gene j form a cis-QTL pair (i.e., SNP l is a cis-QTL for gene j).

The trans-QTL relations are encoded in J, also a $q \times p$ binary matrix. J is defined based on I_1 and I_3. Specifically, SNP l and gene j form a trans-QTL pair if and only if SNP l is a cis-QTL for gene j' *and* gene j' has downstream effect on gene j, $j' \neq j$.

The overall truth is encoded in $\mathbb{1}\big((I_1 + J) \geq 1\big)$, again a $q \times p$ binary matrix. We use this matrix as the truth when calculating AUPRCs. The lj-th entry being one means that SNP l and gene j form a cis-QTL or trans-QTL pair (or both).

	Simulation Design 1	Simulation Design 2
Data simulation	Follows Stegle et al. [1]	Loosely based on Wang et al. [3]
# of experiments	1	176
# of replicates per experiment	10	2
# of simulated data sets	10	352
Genotype data	Simulated (no LD, high MAF)	Real genotype data from GTEx [2]
Cis-QTL relations present	✓	✓
Trans-QTL relations present	✓	✗
Source(s) of expression variation	Primarily trans-regulatory effects	Carefully controlled genotype effects and covariate effects
# of individuals	$n = 80$	$n = 838$
# of genes	$p = 400$	$p = 1{,}000$
# of SNPs	$q = 100$ SNPs in the entire genome	$q = 1{,}000$ local common SNPs per gene
# of known covariates	$K_1 = 3$	$K_1 = 2,\ 3,\ 5,$ or 8 depending on the experiment
# of hidden covariates	$K_2 = 7$	$K_2 = 3,\ 7,\ 15,$ or 22 depending on the experiment

Table S1: Summary of the main differences between Simulation Design 1 and Simulation Design 2. Highlighted in blue are the major data simulation limitations (Section S1) of Simulation Design 1, all of which we address in Simulation Design 2.

Replicate	$\mathrm{Var}(Y_{\mathrm{beforeDSE}})$	$\mathrm{Var}(Y_{\mathrm{DSE}})$	$\mathrm{Var}(Y)$	$\mathrm{Var}(Y_{\mathrm{beforeDSE}}) / \mathrm{Var}(Y)$	$\mathrm{Var}(Y_{\mathrm{DSE}}) / \mathrm{Var}(Y)$
1	124.34	1757.07	1889.57	6.58%	92.99%
2	140.92	1505.17	1677.64	8.40%	89.72%
3	213.56	929.96	1169.18	18.27%	79.54%
4	71.85	761.01	855.39	8.40%	88.97%
5	123.07	2434.45	2574.51	4.78%	94.56%
6	74.94	1029.29	1092.65	6.86%	94.20%
7	148.61	2490.72	2628.93	5.65%	94.74%
8	79.36	796.55	868.05	9.14%	91.76%
9	54.62	1340.10	1390.72	3.93%	96.36%
10	65.64	831.89	895.90	7.33%	92.86%
Average				7.93%	**91.57%**

Table S2: In Simulation Design 1, which follows the data simulation in Stegle et al. [1] as closely as possible, the gene expression levels are primarily driven by trans-regulatory effects rather than cis-regulatory effects or covariate effects. $\mathrm{Var}(Y_{\mathrm{beforeDSE}})$ is defined as the variance of the $n \times p$ entries of $Y_{\mathrm{beforeDSE}}$, and the other variances in the table are defined similarly. We find that $\mathrm{Var}(Y_{\mathrm{DSE}}) / \mathrm{Var}(Y)$ is above 90% on average.

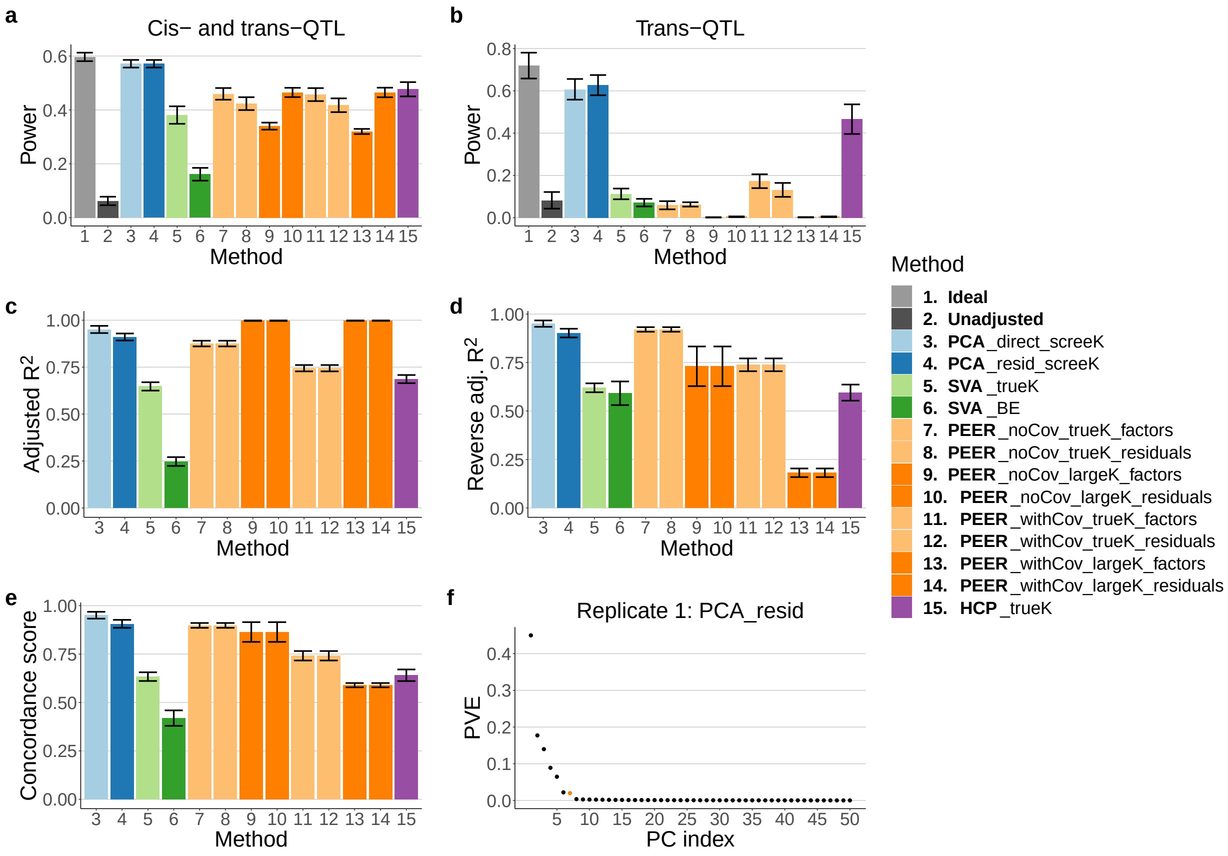

Figure S1: Comparison of all 15 methods (Table 1) in terms of power and adjusted R^2 measures in Simulation Design 1 (the height of each bar represents the average across simulated data sets) and an example scree plot. **a, b** PCA is more powerful than SVA, PEER, and HCP both when we consider all QTL relations (**a**) and when we focus on trans-QTL relations (**b**). Binary decisions are made based on p-values using the Benjamini-Hochberg (BH) procedure and a target false discovery rate of 0.05. **c, d, e** PCA performs the best in terms of concordance score. PEER with a large K (dark orange bars) performs well in terms of adjusted R^2 but less well in terms of reverse adjusted R^2. **f** An example scree plot that unambiguously suggests the true number of hidden covariates, seven in this case, as the reasonable number of PCs to choose (the y-axis represents the proportion of variance explained).

S3 Simulation Design 2

S3.1 Data simulation

In Simulation Design 2, we use real genotype data from GTEx [2], focus on cis-QTL detection, and carefully control the genotype effects and covariate effects in 176 experiments with two replicates per experiment. This simulation design takes inspiration from and is loosely based on Wang et al. [3].

In each experiment-replicate combination, we simulate a data set with $n = 838$ individuals, $p = 1{,}000$ genes, $q = 1{,}000$ local common SNPs per gene, K_1 known covariates, and K_2 hidden covariates (the values of K_1 and K_2 depend on the experiment; see below). Again, let i, j, l, and k be the indices of individuals, genes, SNPs, and covariates, respectively. That is, $i = 1, \cdots, n$; $j = 1, \cdots, p$; $l = 1, \cdots, q$; and $k = 1, \cdots, (K_1 + K_2)$.

We begin by obtaining *SArray*, the $n \times q \times p$ genotype array that remains constant throughout Simulation Design 2. $SArray[, , j]$, an $n \times q$ matrix, is the genotype matrix for the q local common SNPs for gene j. We obtain *SArray* with the following steps:

(a) Download the whole genome sequencing (WGS) phased genotype data for $n = 838$ individuals from GTEx V8 [2].
(b) Randomly select $p = 1{,}000$ genes from the more than 20,000 genes on Chromosomes 1 to 22.
(c) For each gene, obtain the genotype data for the $q = 1{,}000$ SNPs with MAF$\geq$ 0.01 that are the closest to the gene's transcription start site (TSS); we find that these SNPs are almost always within 1 Mb of the TSS. The average MAF of *SArray*, calculated as $\overline{SArray}/2$, the average of all entries of *SArray* divided by 2, is $0.1474385 \approx 0.15$.

Each experiment is characterized by four attributes:

(a) Number of effect SNPs per gene (`numOfEffectSNPs`): 1 or `random`.
(b) Number of covariates (`numOfCovariates`): 5, 10, 20, or 30.
  - Number of known covariates (K_1): 2, 3, 5, 8, respectively.
  - Number of hidden covariates (K_2): 3, 7, 15, 22, respectively.
(c) Proportion of variance explained by genotype (`PVEGenotype`): 0.05, 0.1, 0.2, or 0.3.
(d) Proportion of variance explained by covariates (`PVECovariates`): minimum 0.3, maximum $1 - 0.05 -$ `PVEGenotype`, in increments of 0.1. For example, when `PVEGenotype` $= 0.05$, `PVECovariates` takes seven possible values: 0.3, 0.4, 0.5, $\cdots$, 0.9.

Therefore, we have a total of $2 \times 4 \times (7 + 6 + 5 + 4) = 8 \times 22 = 176$ experiments covering typical scenarios in QTL studies [3]. Following Wang et al. [3], we use the term "effect SNPs" to refer to SNPs that have a nonzero cis effect on a given gene.

Given `numOfEffectSNPs`, `numOfCovariates`, `PVEGenotype`, and `PVECovariates`, we simulate each data set based on

$$\underset{n\times p}{Y} = \underset{n\times q\times p}{SArray} \otimes \left(\underset{q\times p}{I} \odot \underset{q\times p}{B_1} \right) + \underset{n\times(K_1+K_2)}{X} \underset{(K_1+K_2)\times p}{B_2} + \underset{n\times p}{E}, \tag{S4}$$

where Y is the gene expression matrix, and $\otimes$ is defined as

$$\underset{n\times p}{C} = \underset{n\times q\times p}{A} \otimes \underset{q\times p}{B} \quad \Leftrightarrow \quad \underset{n\times 1}{C[, j]} = \underset{n\times q}{A[, , j]} \times \underset{q\times 1}{B[, j]}, \; j = 1, \cdots, p. \tag{S5}$$

Specifically, in the genotype component, we have

- *SArray*: genotype array. $SArray[,,j]$, an $n \times q$ matrix, is the genotype matrix for the q local common SNPs for gene j (see above).
- I: effect indicator matrix.
  - If `numOfEffectSNPs` $= 1$, then for each column, we randomly assign one entry to be one while keeping the other entries zero.
  - If `numOfEffectSNPs` $=$ `random`, then each entry of I is drawn from $Ber(1/q)$. This means that for each gene, the number of effect SNPs is drawn from $Binom(q, \text{prob} = 1/q)$. This binomial distribution approximates the empirical distribution of the number of independent cis-eQTLs per gene in GTEx data [2] well (Figure S2).
- B_1: effect size matrix. Each entry is drawn from $N(0,1)$.

In the covariate component, we have

- X: covariate matrix. Each entry is drawn from $N(0,1)$. As in Simulation Design 1, the first K_1 columns are designated as the known covariates (X_1, $n \times K_1$), and the last K_2 columns are designated as the hidden covariates (X_2, $n \times K_2$).
- B_2: effect size matrix. Each entry is drawn from $N(0,1)$ and scaled (see below).

Lastly, in the noise component, we have

- E: noise matrix. Each entry is drawn from $N(0,1)$ and scaled (see below).

Alternatively, (S4) can be written as

$$\underset{n\times 1}{(Y)_j} = \underset{n\times q}{S_j}\ \underset{q\times 1}{(IB_1)_j} + \underset{n\times(K_1+K_2)}{X}\ \underset{(K_1+K_2)\times 1}{(B_2)_j} + \underset{n\times 1}{(E)_j},\ j = 1,\cdots,p, \tag{S6}$$

where $(Y)_j$, $(IB_1)_j$, $(B_2)_j$, and $(E)_j$ denote the jth column of Y, $I \odot B_1$, B_2, and E, respectively, and S_j denotes $SArray[,,j]$.

The scaling for B_2 and E is to ensure that `PVEGenotype` and `PVECovariates` are as desired. Specifically, for gene j, if $\text{Var}\left(S_j\ (IB_1)_j\right) \neq 0$, then we scale $(B_2)_j$ so that

$$\frac{\text{Var}\left(X\ (B_2)_j\right)}{\text{Var}\left(S_j\ (IB_1)_j\right)} = \frac{\texttt{PVECovariates}}{\texttt{PVEGenotype}} \tag{S7}$$

and separately scale $(E)_j$ so that

$$\frac{\text{Var}\left((E)_j\right)}{\text{Var}\left(S_j\ (IB_1)_j\right)} = \frac{1 - \texttt{PVEGenotype} - \texttt{PVECovariates}}{\texttt{PVEGenotype}}. \tag{S8}$$

If $\mathrm{Var}\left(S_j\ (IB_1)_j\right) = 0$ (which is the case when $(IB_1)_j$ is a zero vector, i.e., when gene j has zero effect SNPs), then we only scale $(E)_j$ so that

$$\frac{\mathrm{Var}\left((E)_j\right)}{\mathrm{Var}\left(X\ (B_2)_j\right)} = \frac{1 - \texttt{PVECovariates}}{\texttt{PVECovariates}}. \tag{S9}$$

S3.2 Definition of truth

In a simulated data set, I is a $q \times p$ binary matrix. The lj-th entry being one means that the lth local common SNP for gene j is an effect SNP for gene j. However, due to LD, the expression level of a gene may be strongly associated with SNPs other than its effect SNPs.

Therefore, we define I_{cor}, also a $q \times p$ binary matrix, based on *SArray* and I and use it as the truth when calculating AUPRCs. The lj-th entry of I_{cor} is defined as one if and only if the lth local common SNP for gene j is highly correlated with *any* of gene j's effect SNPs (correlation ≥ 0.9 in absolute value).

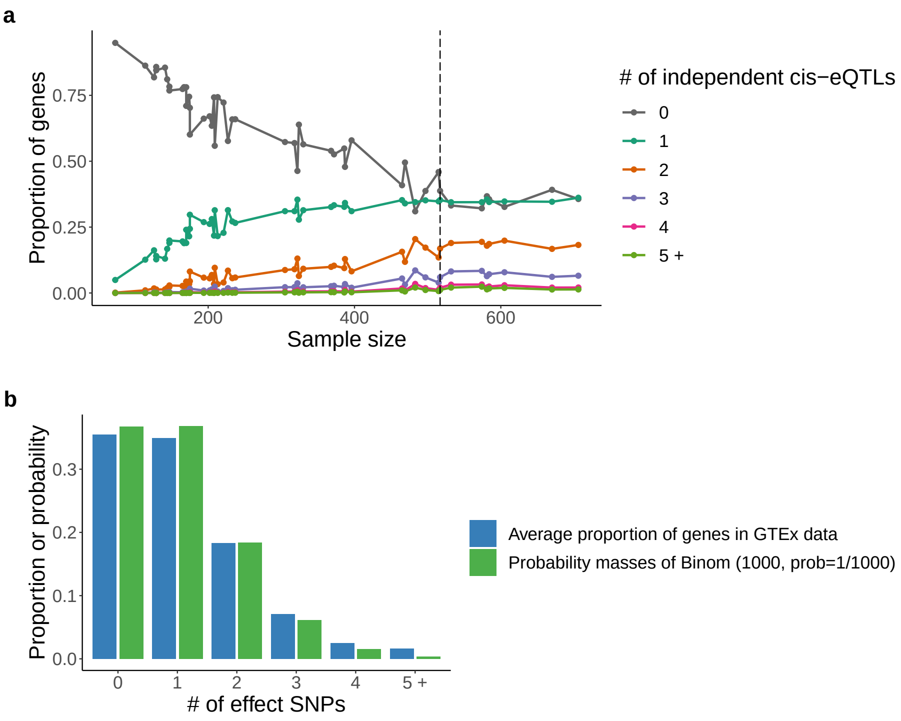

Figure S2: In Simulation Design 2, we find that $\mathit{Binom}(1000, \text{prob} = 1/1000)$ approximates the empirical distribution of the number of independent cis-eQTLs per gene in GTEx data [2] well. **a** Given a tissue type, which corresponds to a sample size, we plot the proportion of genes with 0, 1, 2, 3, 4, or 5 or more independent cis-eQTLs (the proportions add up to one; data from GTEx [2]). We find that the proportions stabilize once the sample size reaches about 517 (dashed line). **b** For the eight tissue types with sample size ≥ 517, we take the average proportion of genes with 0 independent cis-eQTLs, 1 independent cis-eQTL, etc. and plot them in the blue bars. The green bars represent the probability mass function of $\mathit{Binom}(1000, \text{prob} = 1/1000)$ (with the tail probabilities combined together).

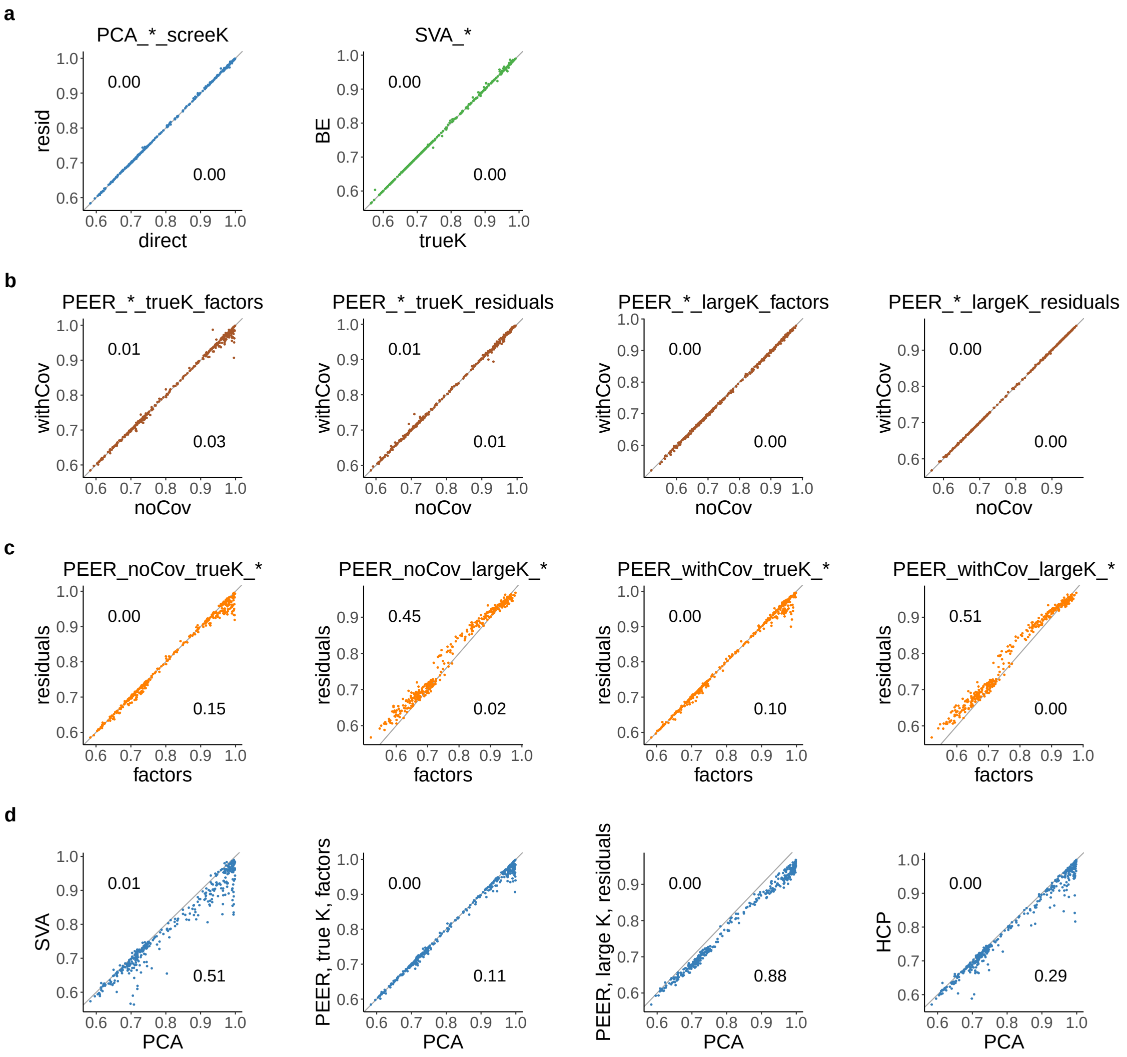

Figure S3: This figure shows how we select a few representative methods from the 15 methods for detailed comparison in Simulation Design 2 (**a, b, c**) and a dataset-by-dataset comparison of the selected representative methods (**d**). The x-axis and y-axis both represent AUPRCs of different methods. Each scatter plot contains 352 points, each of which corresponds to a simulated data set in Simulation Design 2. The number on the upper-left corner of each scatter plot represents the proportion of points that satisfy $y > 1.02\,x$, and the number on the lower-right corner represents the proportion of points that satisfy $x > 1.02\,y$, where x and y denote the coordinates of each point. **a** The two PCA methods perform almost identically, so for simplicity, we select PCA_direct_screeK. The two SVA methods perform almost identically as well, so we select SVA_BE. **b** Whether the known covariates are inputted when PEER is run has little effect on the AUPRC. **c** When we use the true K, the factor approach outperforms the residual approach, but when we use a large K, the residual approach outperforms the factor approach. Therefore, we select PEER_withCov_trueK_factors and PEER_withCov_largeK_residuals as the representative PEER methods. **d** Among the selected representative methods, PCA outperforms SVA, PEER, and HCP in terms of AUPRC in 11% to 88% of the simulated data sets and underperforms them in close to 0% of the simulated data sets.

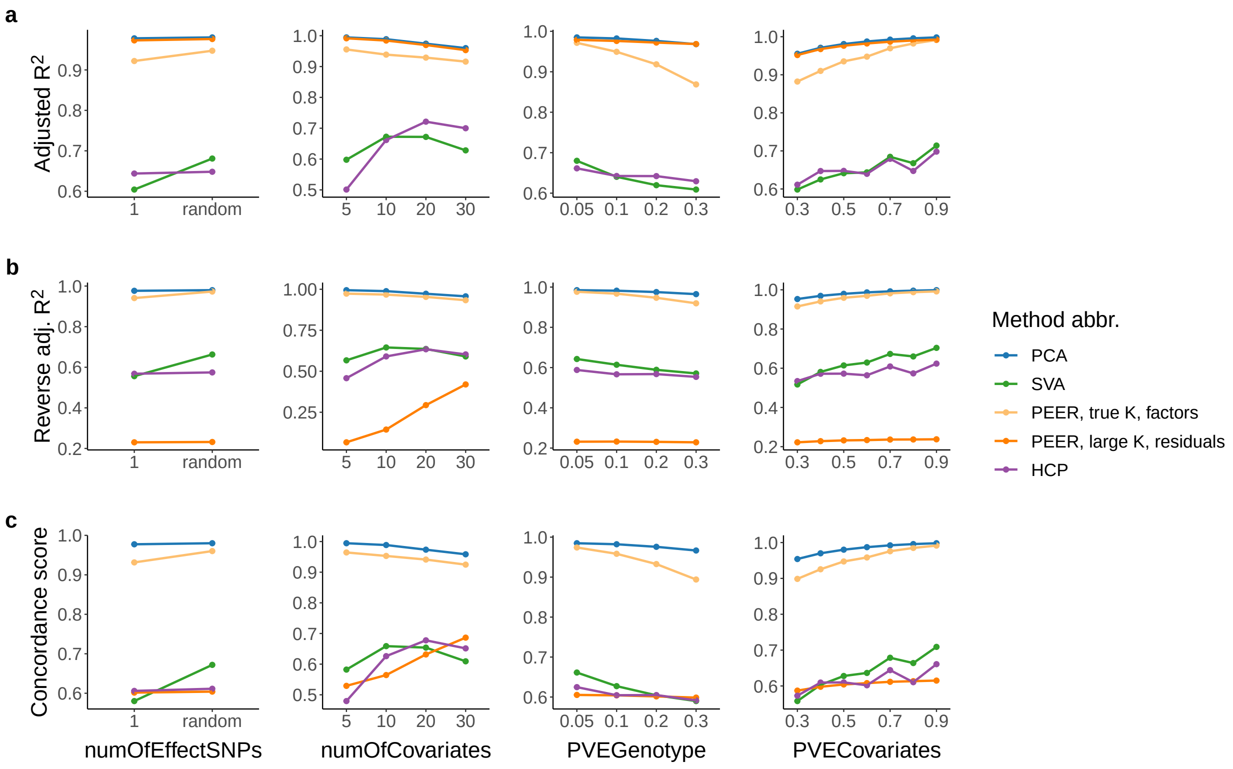

Figure S4: Detailed adjusted R^2, reverse adjusted R^2, and concordance score comparison of the selected representative methods (Table 1) in Simulation Design 2. Each point represents the average across simulated data sets. PCA performs the best in all three regards. PEER with a large K (dark orange line) performs well in terms of adjusted R^2 but falls short in terms of reverse adjusted R^2.

S4 Compared methods

We compare the runtime and performance of 15 methods based on simulation studies, including Ideal, Unadjusted, and 13 variants of PCA, SVA, PEER, and HCP (Table 1). The details of Simulation Design 1 and Simulation Design 2 are described in Sections S2 and S3, respectively. Recall that in each simulated data set, Y denotes the gene expression matrix ($n \times p$, sample by gene), X_1 denotes the known covariate matrix ($n \times K_1$, sample by covariate), and X_2 denotes the hidden covariate matrix ($n \times K_2$, sample by covariate). The genotype information is stored in S in Simulation Design 1 and $SArray$ in Simulation Design 2. In this work, we use K to denote the number of inferred covariates, which are called PCs, SVs, PEER factors, and HCPs in PCA, SVA, PEER, and HCP, respectively.

Given a simulated data set, each of the 15 methods consists of two steps: hidden variable inference step (not applicable for Ideal and Unadjusted) and QTL step. In the hidden variable inference step, we run PCA, SVA, PEER, or HCP to obtain the inferred covariates (and the expression residuals in the case of PEER; Figure 1). In the QTL step, given a gene-SNP pair, we

run a linear regression with the gene expression vector (or the residual vector from PEER) as the *response* and the genotype vector and covariates as *predictors*, where the choice of the response and covariates depends on the method (Table 1); thus we obtain the p-value for the null hypothesis that the coefficient corresponding to the genotype vector is zero given the covariates. In Simulation Design 1, we investigate the association between each gene's expression level and each SNP in the *entire genome* for a simultaneous detection of cis-QTL and trans-QTL relations. In Simulation Design 2, we investigate the association between each gene's expression level and each of the gene's *local common SNPs* for a cis-QTL analysis.

For Ideal, we assume that X_2 is known. Therefore, we use X_1 and X_2 as covariates in the QTL step. For Unadjusted, we use X_1 as the covariates.

We devise two ways to use PCA to account for the hidden covariates. For PCA_direct_screeK, we run PCA on Y directly. For PCA_resid_screeK, we first residualize Y against X_1 and then run PCA on the residual matrix. In this work, PCA is run *with* centering and scaling unless otherwise specified; given A, an $n \times p_1$ matrix, and B, an $n \times p_2$ matrix, both observation by feature, to *residualize* A *against* B means to take each column of A, regress it against B, and replace the original column of A with the residuals from the linear regression. For both methods, since the scree plots always suggest the true "number of hidden covariates" ($K_1 + K_2$ for PCA_direct_screeK, K_2 for PCA_resid_screeK) as the reasonable number of PCs to choose within plus or minus one (usually exactly; Figure S1), we set the number of PCs to be the true "number of hidden covariates". For PCA_direct_screeK, we filter out the known covariates that are captured well by the top PCs (unadjusted $R^2 \geq 0.9$) and use the remaining known covariates along with the top PCs as covariates in the QTL step. For PCA_resid_screeK, no filtering is needed.

Here we describe the two hidden variable inference methods for SVA: SVA_trueK and SVA_BE. Since the SVA package [4] requires the user to input at least one variable of interest (Figure 1) and using too many variables of interest causes the package to fail, when running SVA, we input the top PC of the genotype matrix (S in Simulation Design 1, collapsed version of $SArray$ in Simulation Design 2) as the variable of interest. We also input X_1 as the known covariates because the package documentation indicates that the known covariates should be provided if available. The SVA package allows the user to specify K. Alternatively, it can automatically choose K using a slightly modified version of the Buja and Eyuboglu (BE) algorithm [5, 6]. Therefore, in SVA_trueK, we set $K = K_2$, and in SVA_BE, we let the package choose K automatically. In both cases, we use X_1 and the surrogate variables (SVs) as covariates in the QTL step.

There are several different ways to use PEER [7] but no consensus in the literature on which one is the best. In the hidden variable inference step, PEER can be run with or without the known covariates when there are known covariates available (Stegle et al. [7] do not give an explicit recommendation as to which approach should be used, and both approaches are used in practice [2, 8–10]), and K has to be specified by the user (Stegle et al. [1, 7] claim that the performance of PEER does not deteriorate as K increases). In the QTL step, one can include the PEER factors as covariates (we call this the "factor approach") or use the expression residuals outputted by PEER as the response (and not use any known or inferred covariates; we call this the "residual approach"). For completeness, we compare $2^3 = 8$ ways of using PEER (the default priors are always used):

PEER is run with or without the known covariates; PEER is run using the true "number of hidden covariates" ($K_1 + K_2$ when PEER is run without the known covariates, K_2 when PEER is run with the known covariates) or using a large K (K=50); and either the factor approach or the residual approach is used in the QTL step.

The HCP package requires the user to specify K and three tuning parameters: λ_1, λ_2, and λ_3 (Section S5.2). The package documentation suggests choosing K and the tuning parameters via a grid search. However, no specific recommendations are given regarding the choice of the score function. In practice, users of HCP often choose K and the tuning parameters by maximizing the number of discoveries [11, 12]. For our simulation studies, such an approach would be computationally prohibitive. Therefore, for simplicity, we set $K = K_2$ and $\lambda_1 = \lambda_2 = \lambda_3 = 1$; the latter is because we do not want to give more weight to the penalty terms than the main term in the objective function (Section S5.2).

**Reference**	**GTEx data version**	**QTL analysis**	**Data transformation**	**Known covariates inputted**	**# of PEER factors**	**Factor or residual approach**
(A)	(B)	(C)	(D)	(E)	(F)	(G)
GTEx Consortium [8]	V6p	eQTL (cis and trans)	INT within feature	No	Maximizes cis-eGenes	Factor
GTEx Consortium [2]	V8	eQTL (cis and trans)	INT within feature	No	Maximizes cis-eGenes	Factor
		sQTL (cis and trans)	INT within sample	No	15	Factor
Li et al. [9]	V7	$3'$aQTL (cis)	No transformation	Yes	Follows GTEx [8]	Factor

Table S3: Summary of QTL analyses performed by GTEx [2, 8] and Li et al. [9]. "INT" in (D) stands for "inverse normal transform" [13]. (E), (F), and (G) summarize how PEER is used (Section S4) in each study. GTEx [2, 8] chooses the number of PEER factors for its eQTL analyses (including cis and trans) by maximizing the number of discovered cis-eGenes for each pre-defined sample size bin. The number of PEER factors selected is 15 for $n < 150$, 30 for $n \in [150, 250)$, and 35 for $n \geq 250$ for GTEx V6p eQTL analyses [8] and 15 for $n < 150$, 30 for $n \in [150, 250)$, 45 for $n \in [250, 350)$, and 60 for $n \geq 350$ for GTEx V8 eQTL analyses [2], where n denotes the sample size. Li et al. [9] use the numbers of PEER factors chosen by GTEx [8].

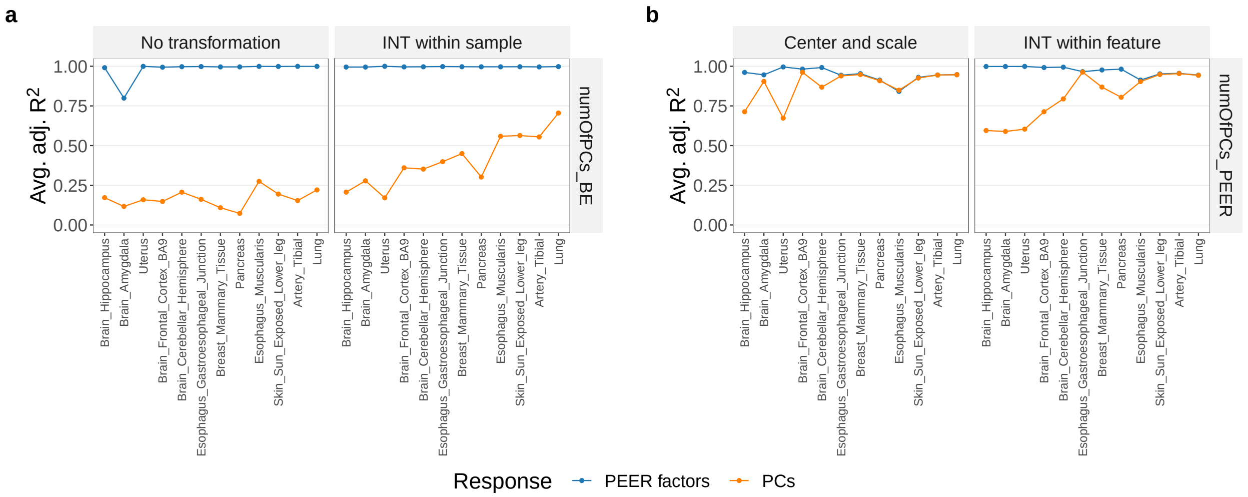

Figure S5: In the 3′aQTL data prepared by Li et al. [9] from GTEx RNA-seq reads [8], PEER factors fail to capture important variance components of the molecular phenotype data when the data transformation method is "No transformation" or "INT within sample" (**a**; the numbers of PCs are chosen via BE (Algorithm S3)). On the other hand, PEER factors span roughly the same linear subspace as the top PCs when the data transformation method is "Center and scale" or "INT within feature", but the top PCs can almost always capture the PEER factors better than the PEER factors can capture the top PCs (**b**; the numbers of PCs are equal to the numbers of PEER factors). Given m PEER factors and n PCs from the same post-transformation molecular phenotype matrix ($m \geq n$ in **a**, $m = n$ in **b**), we calculate m adjusted R^2's by regressing each PEER factor against the PCs and plot the average in blue. Similarly, we calculate n adjusted R^2's by regressing each PC against the PEER factors and plot the average in orange.

Algorithm S1: Reordering of PEER factors based on PCs (Figure 5).

Inputs:

- K PEER factors.
- K PCs.

Output: K PEER factors (reordered).

for $k \leftarrow 1$ **to** K **do**
    Select the PEER factor that is the most highly correlated with the kth PC from the PEER factors that have not been selected yet.
end
return the PEER factors in the order that they were selected in.

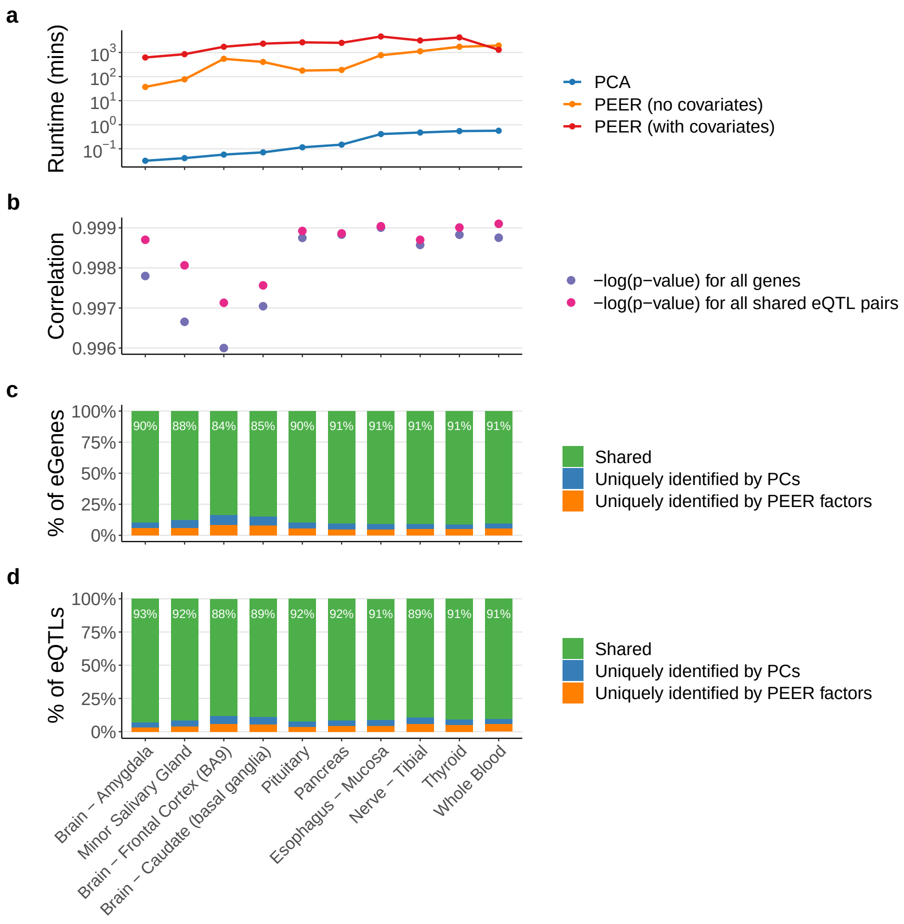

Figure S6: In GTEx eQTL data [2], PEER is at least three orders of magnitude slower than PCA (**a**), and replacing the PEER factors with PCs in GTEx's FastQTL pipeline [2, 14] does not change the cis-eQTL results much (**b, c, d**). The x-axis shows 10 randomly selected tissue types with increasing sample sizes. **a** For a given gene expression matrix, running PEER without the known covariates (GTEx's approach) takes up to about 1,900 minutes (equivalent to about 32 hours; Whole Blood), while running PCA (with centering and scaling; our approach) takes no more than a minute. For comparison, we also run PEER *with* the known covariates using the numbers of PEER factors selected by GTEx. This approach takes even longer (up to about 4,600 minutes, equivalent to about 77 hours; Esophagus - Mucosa). **b** The p-values produced by GTEx's approach and our approach are highly correlated (correlations between the negative common logarithms are shown). **c, d** The overlap of the identified eGenes and eQTL pairs between the two approaches is generally around 90% (see Figure S7 for more detail).

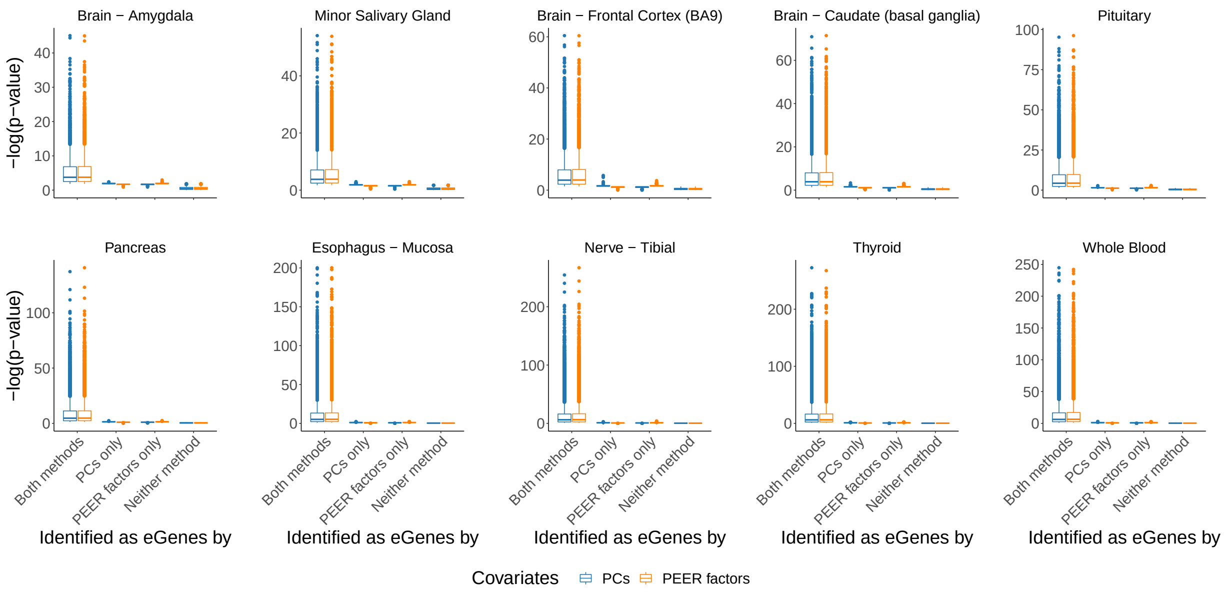

Figure S7: Following the analysis in Figure S6, we find that the eGenes uniquely identified by PCs or PEER factors have marginal p-values compared to those identified by both methods.

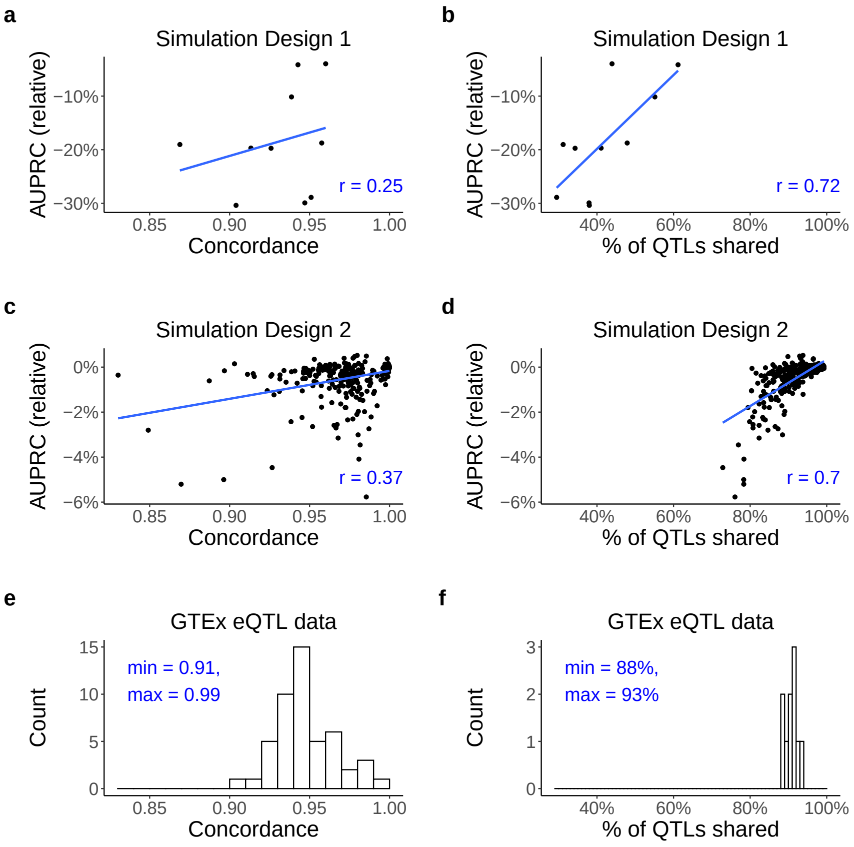

Figure S8: Joint analysis of results from Simulation Design 1, Simulation Design 2, and GTEx eQTL data [2]. The x-axes of **a**, **c**, and **e** show the concordance between PEER factors and top PCs (defined analogously as the concordance score; Methods). The x-axes of **b**, **d**, and **f** show the percentage of QTL discoveries shared between PEER and PCA (in **b** and **d**, for each method, binary decisions are made based on p-values using the Benjamini-Hochberg (BH) procedure and a target false discovery rate of 0.05). In **a** through **d**, the y-axes show $(\text{AUPRC}_{\text{PEER}} - \text{AUPRC}_{\text{PCA}})/\text{AUPRC}_{\text{PCA}}$, the blue lines are the simple linear regression lines, and the Pearson correlation coefficients are shown on the bottom right. **a** and **b** each contains 10 data points, corresponding to the 10 simulated data sets in Simulation Design 1. **c** and **d** each contains 352 data points, corresponding to the 352 simulated data sets in Simulation Design 2. The methods compared in **a** through **d** are PCA_direct_screeK and PEER_noCov_trueK_factors. **e** presents similar information as Figure 5; the total count is 49, which is the number of tissue types with GTEx eQTL analyses. **f** is based on Figure S6(d); the total count is 10, which is the number of tissue types randomly selected for analysis in Figure S6. We find that the percentage of QTL discoveries shared is a good predictor of the relative performance of PEER versus PCA and is a better predictor than concordance. This plot is also evidence that Simulation Design 2 is more realistic than Simulation Design 1 because the ranges that concordance and percentage of QTL discoveries shared fall in in **e** and **f** agree better with those in **c** and **d** than those in **a** and **b**.

S5 Theory of PCA and HCP

S5.1 Principal component analysis (PCA)

Principal component analysis (PCA) [15, 16] is a well-established dimension reduction method with many applications. Here we aim to provide a brief summary of its algorithm, derivation, and interpretation.

Let X denote the $n \times p$ observed data matrix that is observation by feature, e.g., a molecular phenotype matrix. We use X instead of Y here to be consistent with standard PCA notations. We assume that the columns of X have been centered and scaled. That is, X satisfies

$$\frac{1}{n}\sum_{i=1}^{n} x_{ij} = 0\,,\ j = 1, \cdots, p \tag{S10}$$

and

$$\frac{1}{n-1}\sum_{i=1}^{n} x_{ij}^2 = 1\,,\ j = 1, \cdots, p\,, \tag{S11}$$

where x_{ij} denotes the ij-th entry of X.

The PCA algorithm consists of two steps. In the first step, we calculate the sample covariance matrix $\widehat{\Sigma}$ and perform eigendecomposition on it:

$$\begin{aligned} \widehat{\Sigma} &= \frac{1}{n} X^\top X && \text{definition of sample covariance matrix} && \text{(S12)} \\ &:= Q \Lambda Q^\top\,, && \text{eigendecomposition} && \text{(S13)} \end{aligned}$$

where

$$\underset{p\times p}{Q} = \begin{bmatrix} | & & | \\ q_1 & \cdots & q_p \\ | & & | \end{bmatrix} \tag{S14}$$

is an orthogonal matrix whose columns are eigenvectors of $\widehat{\Sigma}$, and

$$\underset{p\times p}{\Lambda} = \begin{bmatrix} \lambda_1 & & \\ & \ddots & \\ & & \lambda_p \end{bmatrix}\,,\ \lambda_1 \geq \cdots \geq \lambda_p \geq 0\,, \tag{S15}$$

is a diagonal matrix whose diagonal entries are the corresponding eigenvalues of $\widehat{\Sigma}$. We know that $\widehat{\Sigma}$ is orthogonally diagonalizable because it is a symmetric matrix (recall the spectral theorem for real matrices [17]: a real matrix is orthogonally diagonalizable if and only if it is symmetric). The eigenvalues are all non-negative because $\widehat{\Sigma}$ is positive semidefinite.

In the second step, we calculate Z as

$$Z = XQ\,, \tag{S16}$$

where the columns of Z are called the *principal components* (PCs) or *scores*, and Q is called the *loading matrix* or *rotation matrix*. It is worth noting that some authors may refer to $q_1, \cdots, q_p$ as the PCs. This use of terminology is confusing and should be avoided [18].

The above two steps conclude the PCA algorithm. In practice, however, singular value decomposition (SVD) of the data matrix is often used as a more computationally efficient way of finding the loading matrix and the PCs [15].

The most common derivation of PCA is based on maximum variance [19]. First, we define $\alpha_1^*, \cdots, \alpha_p^* \in \mathbb{R}^p$ sequentially as

$$\alpha_1^* = \underset{\alpha_1 \in \mathbb{R}^p}{\arg\max} \operatorname{Var}(X\alpha_1) \qquad \text{subject to } \|\alpha_1\|_2 = 1\,, \tag{S17}$$

$$\alpha_2^* = \underset{\alpha_2 \in \mathbb{R}^p}{\arg\max} \operatorname{Var}(X\alpha_2) \qquad \text{subject to } \|\alpha_2\|_2 = 1\,,\ \alpha_2^\top \alpha_1^* = 0\,, \tag{S18}$$

$$\vdots$$

$$\alpha_p^* = \underset{\alpha_p \in \mathbb{R}^p}{\arg\max} \operatorname{Var}(X\alpha_p) \qquad \text{subject to } \|\alpha_p\|_2 = 1\,,\ \alpha_p^\top \alpha_j^* = 0\ \forall j < p\,. \tag{S19}$$

Then, we define the PCs of X as $X\alpha_1^*, \cdots, X\alpha_p^*$. That is, the PCs are defined sequentially as the linear combinations of the columns of X with maximum variances, subject to certain constraints. It can then be shown that $\alpha_1^*, \cdots, \alpha_p^*$ are given by $q_1, \cdots, q_p$ respectively, where $q_1, \cdots, q_p$ are eigenvectors of $\widehat{\Sigma}$ as defined in (S14).

A complementary property of PCA, which is closely related to the original discussion of Pearson [20], is the minimum reconstruction error property. Given $K < p$, we define Q_K as the matrix that contains the first K columns of Q. That is,

$$\underset{p \times K}{Q_K} := \begin{bmatrix} | & & | \\ q_1 & \cdots & q_K \\ | & & | \end{bmatrix}. \tag{S20}$$

The minimum reconstruction error property of PCA states that Q_K is a global minimizer of the loss

function

$$\mathscr{J}\left(\widetilde{Q}_K\right) := \left\|\left| X - X\widetilde{Q}_K\widetilde{Q}_K^\top \right|\right\|_F^2 \tag{S21}$$

$$= \sum_{i=1}^{n} \left\| x_i^\top - x_i^\top \widetilde{Q}_K\widetilde{Q}_K^\top \right\|_2^2 = \sum_{i=1}^{n} \left\| x_i - \widetilde{Q}_K\widetilde{Q}_K^\top x_i \right\|_2^2 , \tag{S22}$$

where $\widetilde{Q}_K$ denotes an arbitrary $p \times K$ matrix whose columns are orthonormal, $|||\cdot|||_F$ denotes the Frobenius norm of a matrix, and $x_i^\top$ denotes the ith row of X. Since $\widetilde{Q}_K\widetilde{Q}_K^\top x_i$ represents the (orthogonal) projection of x_i onto the subspace spanned by the columns of $\widetilde{Q}_K$, (S22) measures the total squared ℓ_2 error when approximating each x_i with its projection onto the subspace spanned by the columns of $\widetilde{Q}_K$.

A central idea of PCA is the proportion of variance explained by each PC. To establish this concept, we claim that

$$\sum_{j=1}^{p} \operatorname{Var}\left(X_j\right) = \sum_{j=1}^{p} \operatorname{Var}\left(Z_j\right) , \tag{S23}$$

$$\operatorname{Var}\left(Z_j\right) = \lambda_j ,\ j = 1, \cdots , p, \tag{S24}$$

and

$$\operatorname{Cov}\left(Z_j\, ,\, Z_{j'}\right) = 0 ,\ j, j' = 1, \cdots , p,\ j \neq j' , \tag{S25}$$

where X_j denotes the jth column of X (the jth original variable) and Z_j denotes the jth column of Z (the jth PC). (S25) means that the PCs are uncorrelated with each other.

We prove (S24) and (S25) by calculating $\widehat{\Sigma}_Z$, the sample covariance matrix of Z (we know that the columns of Z are centered by (S10) and (S16)):

$$\widehat{\Sigma}_Z = \frac{1}{n} Z^\top Z \qquad \text{definition of sample covariance matrix} \tag{S26}$$

$$= \frac{1}{n} (XQ)^\top XQ \qquad \text{plugging in (S16)} \tag{S27}$$

$$= Q^\top \left(\frac{1}{n} X^\top X\right) Q \tag{S28}$$

$$= Q^\top \left(Q\Lambda Q^\top\right) Q \qquad \text{plugging in (S13)} \tag{S29}$$

$$= \Lambda . \tag{S30}$$

(S23) can be proven by the following:

$$
\begin{aligned}
\sum_{j=1}^{p} \operatorname{Var}\left(X_j\right) &= \operatorname{Tr}\left(\widehat{\Sigma}\right) && \text{definition of trace and } \widehat{\Sigma} && \text{(S31)}\\
&= \operatorname{Tr}\left(Q\Lambda Q^\top\right) && \text{plugging in (S13)} && \text{(S32)}\\
&= \operatorname{Tr}\left(\Lambda Q^\top Q\right) && \text{cyclic property of trace} && \text{(S33)}\\
&= \operatorname{Tr}\left(\Lambda\right) && && \text{(S34)}\\
&= \sum_{j=1}^{p} \operatorname{Var}\left(Z_j\right). && \text{by (S24)} && \text{(S35)}
\end{aligned}
$$

Because of (S23) and (S24), we may define the proportion of variance in the original data explained by the jth PC as

$$
\frac{\lambda_j}{\sum_{j'=1}^{p} \operatorname{Var}\left(X_{j'}\right)} = \frac{\lambda_j}{\sum_{j'=1}^{p} \operatorname{Var}\left(Z_{j'}\right)} = \frac{\lambda_j}{\sum_{j'=1}^{p} \lambda_{j'}}, \tag{S36}
$$

which provides a basis for deciding the number of PCs to keep (e.g., Algorithms S2 and S3).

S5.2 Hidden covariates with prior (HCP) and its connection to PCA

Hidden covariates with prior (HCP) [21] is a popular hidden variable inference method for QTL mapping defined by minimizing a loss function. Neither Mostafavi et al. [21] nor the HCP package documents the HCP method well. For example, the squares in the loss function (S37) are missing in both Mostafavi et al. [21] and the package documentation, but one can deduce that the squares are there by inspecting the coordinate descent steps in the source code of the R package. Here we aim to provide a better, more accurate documentation of the HCP method and point out its connection to PCA.

Given Y, the molecular phenotype matrix ($n \times p$, sample by feature), X_1, the known covariate matrix ($n \times K_1$, sample by covariate), K, the number of inferred covariates (HCPs), and $\lambda_1,\ \lambda_2,\ \lambda_3 > 0$, the tuning parameters, HCP looks for

$$
\underset{X_2,\, W_1,\, W_2}{\arg\min} \left\{ \left\|\left\| \underset{n\times p}{Y} - \underset{n\times K}{X_2}\ \underset{K\times p}{W_2} \right\|\right\|_F^2 + \lambda_1 \left\|\left\| \underset{n\times K}{X_2} - \underset{n\times K_1}{X_1}\ \underset{K_1\times K}{W_1} \right\|\right\|_F^2 + \lambda_2 |||W_1|||_F^2 + \lambda_3 |||W_2|||_F^2 \right\}, \tag{S37}
$$

where $|||\cdot|||_F$ denotes the Frobenius norm of a matrix, X_2 is the hidden covariate matrix, and W_1 and W_2 are weight matrices of the appropriate dimensions. The name of the method, "hidden covariates with prior", comes from the second term in (S37), where the method informs the hidden

covariates with the known covariates. The optimization is done through coordinate descent with one deterministic initialization (see source code of the HCP R package [21]). The columns of the obtained X_2 are reported as the HCPs.

From (S37), we see that the HCP method is closely related to PCA. The first term in (S37) is very similar to (S21), the only difference being that the rows of W_2 in (S37) are not required to be orthonormal and X_2 is not required to be equal to $YW_2^\top$.

Algorithm S2: The elbow method for choosing K in PCA (based on distance to diagonal line).

Input: X, $n \times p$ observed data matrix, observation by feature.
Output: K, the number of PCs selected.

Define $d = \texttt{min}(n, p)$. This is the total number of PCs.
Run PCA on X with centering and scaling.
Obtain the proportion of variance explained by each PC, $t_1, \cdots, t_d$. // $\sum_{j=1}^{d} t_j = 1$.
Consider $(1, t_1), \cdots, (d, t_d) \in \mathbb{R}^2$. // d points in $\mathbb{R}^2$.
Select K by choosing the point that is the farthest from the diagonal line, i.e., the line that passes through the first point, $(1, t_1)$, and the last point, (d, t_d). Specifically, the distance from (x_0, y_0) to the line that passes through (x_1, y_1) and (x_2, y_2) is given by $|\,(x_2 - x_1)(y_1 - y_0) - (x_1 - x_0)(y_2 - y_1)\,| \,/ ((x_2 - x_1)^2 + (y_2 - y_1)^2)^{1/2}$.
return K.

Algorithm S3: The Buja and Eyuboglu (BE) algorithm [5] for choosing K in PCA.

Inputs:

- X, $n \times p$ observed data matrix, observation by feature.
- B, number of permutations (default is 20).
- α, significance level (default is 0.05).

Output: K, the number of PCs selected.

Define $d = \texttt{min}(n, p)$. This is the total number of PCs.

Run PCA on X with centering and scaling.

Obtain the proportion of variance explained (PVE) by each PC, $t_1, \cdots, t_d$. // $\sum_{j=1}^{d} t_j = 1$. Observed test statistics.

for $b \leftarrow 1$ **to** B **do**

  Obtain $X^{(b)}$ by permuting each column of X. // Permute the observations in each feature.

  Run PCA on $X^{(b)}$ with centering and scaling.

  Obtain the PVE of each PC, $t_1^{(b)}, \cdots, t_d^{(b)}$. // $\sum_{j=1}^{d} t_j^{(b)} = 1$.

end

The p-value for the jth PC is calculated as

$p_j = \left(\sum_{b=1}^{B} \mathbb{1}\{t_j^{(b)} \geq t_j\} + 1\right) / (B+1),\ j = 1, \cdots, d.$ // p_j is calculated as, roughly speaking, the proportion of permutations where the PVE of the jth PC is greater than or equal to the PVE of the jth original PC (the added ones in the numerator and denominator are mainly for avoiding p-values that are exactly zero). The greater this proportion is, the larger the p-value is, and the less significant the PC is.

for $j \leftarrow 2$ **to** d **do**

  If $p_j \leq p_{j-1}$, then set $p_j = p_{j-1}$. // Enforce monotone increase of the p-values.

end

Set K to be the maximum j such that $p_j \leq \alpha$.

return K.

References

[1] Oliver Stegle, Leopold Parts, Richard Durbin, and John Winn. A Bayesian framework to account for complex non-genetic factors in gene expression levels greatly increases power in eQTL studies. *PLoS Computational Biology*, 6(5):e1000770, 2010.

[2] GTEx Consortium. The GTEx Consortium atlas of genetic regulatory effects across human tissues. *Science*, 369(6509):1318–1330, 2020.

[3] Gao Wang, Abhishek Sarkar, Peter Carbonetto, and Matthew Stephens. A simple new approach to variable selection in regression, with application to genetic fine mapping. *Journal of the Royal Statistical Society: Series B (Statistical Methodology)*, 82(5):1273–1300, 2020.

[4] Jeffrey T. Leek, W. Evan Johnson, Hilary S. Parker, Andrew E. Jaffe, and John D. Storey. The sva package for removing batch effects and other unwanted variation in high-throughput experiments. *Bioinformatics*, 28(6):882–883, 2012.

[5] Andreas Buja and Nermin Eyuboglu. Remarks on parallel analysis. *Multivariate Behavioral Research*, 27(4):509–540, 1992.

[6] Heather J. Zhou. Capturing hidden covariates with linear factor models and other statistical methods in differential gene expression and expression quantitative trait locus studies. *UCLA Electronic Theses and Dissertations*, 2022. `https://escholarship.org/uc/item/2rq72420`.

[7] Oliver Stegle, Leopold Parts, Matias Piipari, John Winn, and Richard Durbin. Using probabilistic estimation of expression residuals (PEER) to obtain increased power and interpretability of gene expression analyses. *Nature Protocols*, 7(3):500–507, 2012.

[8] GTEx Consortium. Genetic effects on gene expression across human tissues. *Nature*, 550 (7675):204–213, 2017.

[9] Lei Li, Kai-Lieh Huang, Yipeng Gao, Ya Cui, Gao Wang, Nathan D. Elrod, Yumei Li, Yiling Elaine Chen, Ping Ji, Fanglue Peng, William K. Russell, Eric J. Wagner, and Wei Li. An atlas of alternative polyadenylation quantitative trait loci contributing to complex trait and disease heritability. *Nature Genetics*, 53(7):994–1005, 2021.

[10] Christopher E. Gillies, Rosemary Putler, Rajasree Menon, Edgar Otto, Kalyn Yasutake, Viji Nair, Paul Hoover, David Lieb, Shuqiang Li, Sean Eddy, Damian Fermin, Michelle T. McNulty, Nir Hacohen, Krzysztof Kiryluk, Matthias Kretzler, Xiaoquan Wen, Matthew G. Sampson, John Sedor, Katherine Dell, Marleen Schachere, Kevin Lemley, Lauren Whitted, Tarak Srivastava, Connie Haney, Christine Sethna, Kalliopi Grammatikopoulos, Gerald Appel, Michael Toledo, Laurence Greenbaum, Chia-shi Wang, Brian Lee, Sharon Adler, Cynthia Nast, Janine LaPage, Ambarish Athavale, Alicia Neu, Sara Boynton, Fernando Fervenza, Marie Hogan, John C. Lieske, Vladimir Chernitskiy, Frederick Kaskel, Neelja Kumar, Patricia Flynn, Jeffrey Kopp, Eveleyn Castro-Rubio, Jodi Blake, Howard Trachtman, Olga Zhdanova, Frank Modersitzki, Suzanne Vento, Richard Lafayette, Kshama Mehta,

Crystal Gadegbeku, Duncan Johnstone, Daniel Cattran, Michelle Hladunewich, Heather Reich, Paul Ling, Martin Romano, Alessia Fornoni, Laura Barisoni, Carlos Bidot, Matthias Kretzler, Debbie Gipson, Amanda Williams, Renee Pitter, Patrick Nachman, Keisha Gibson, Sandra Grubbs, Anne Froment, Lawrence Holzman, Kevin Meyers, Krishna Kallem, Fumei Cerecino, Kamal Sambandam, Elizabeth Brown, Natalie Johnson, Ashley Jefferson, Sangeeta Hingorani, Kathleen Tuttle, Laura Curtin, S. Dismuke, Ann Cooper, Barry Freedman, Jen Jar Lin, Stefanie Gray, Matthias Kretzler, Larua Barisoni, Crystal Gadegbeku, Brenda Gillespie, Debbie Gipson, Lawrence Holzman, Laura Mariani, Matthew G. Sampson, Peter Song, Johnathan Troost, Jarcy Zee, Emily Herreshoff, Colleen Kincaid, Chrysta Lienczewski, Tina Mainieri, Amanda Williams, Kevin Abbott, Cindy Roy, Tiina Urv, and John Brooks. An eQTL landscape of kidney tissue in human nephrotic syndrome. *The American Journal of Human Genetics*, 103(2):232–244, 2018.

[11] Rebecca L. Walker, Gokul Ramaswami, Christopher Hartl, Nicholas Mancuso, Michael J. Gandal, Luis de la Torre-Ubieta, Bogdan Pasaniuc, Jason L. Stein, and Daniel H. Geschwind. Genetic control of expression and splicing in developing human brain informs disease mechanisms. *Cell*, 179(3):750–771, 2019.

[12] Alexis Battle, Sara Mostafavi, Xiaowei Zhu, James B. Potash, Myrna M. Weissman, Courtney McCormick, Christian D. Haudenschild, Kenneth B. Beckman, Jianxin Shi, Rui Mei, Alexander E. Urban, Stephen B. Montgomery, Douglas F. Levinson, and Daphne Koller. Characterizing the genetic basis of transcriptome diversity through RNA-sequencing of 922 individuals. *Genome Research*, 24(1):14–24, 2014.

[13] T. Mark Beasley, Stephen Erickson, and David B. Allison. Rank-based inverse normal transformations are increasingly used, but are they merited? *Behavior Genetics*, 39(5):580–595, 2009.

[14] Halit Ongen, Alfonso Buil, Andrew Anand Brown, Emmanouil T. Dermitzakis, and Olivier Delaneau. Fast and efficient QTL mapper for thousands of molecular phenotypes. *Bioinformatics*, 32(10):1479–1485, 2016.

[15] Ian T. Jolliffe. *Principal Component Analysis*. Springer, New York, second edition, 2002.

[16] Richard A. Johnson and Dean W. Wichern. *Applied Multivariate Statistical Analysis*. Pearson Prentice Hall, Upper Saddle River, NJ, sixth edition, 2007.

[17] Otto Bretscher. *Linear Algebra With Applications*. Pearson Prentice Hall, Upper Saddle River, NJ, fourth edition, 2009.

[18] Ian T. Jolliffe and Jorge Cadima. Principal component analysis: A review and recent developments. *Philosophical Transactions of the Royal Society A*, 374(2065), 2016.

[19] Harold Hotelling. Analysis of a complex of statistical variables into principal components. *Journal of Educational Psychology*, 24(6):417–441, 1933.

[20] Karl Pearson. LIII. On lines and planes of closest fit to systems of points in space. *The London, Edinburgh, and Dublin Philosophical Magazine and Journal of Science*, 2(11):559–572, 1901.

[21] Sara Mostafavi, Alexis Battle, Xiaowei Zhu, Alexander E. Urban, Douglas Levinson, Stephen B. Montgomery, and Daphne Koller. Normalizing RNA-sequencing data by modeling hidden covariates with prior knowledge. *PLoS ONE*, 8(7):e68141, 2013.
