# Supplementary material for: PCA outperforms popular hidden variable inference methods for molecular QTL mapping
Source: Genome Biol. 2022 Oct 11;23:210. doi: 10.1186/s13059-022-02761-4 (PMC9552461; doi:10.1186/s13059-022-02761-4)
Supplement: Supplementary file 2 — Additional file 2: Review history. [file 13059_2022_2761_MOESM2_ESM.docx]

Review History

**First round of review**

**Reviewer 1**

**Were you able to assess all statistics in the manuscript, including the appropriateness of statistical tests used?**

Yes

**Comments to author:**

The authors compared 15 methods of hidden variable inference for eQTL mapping. These methods are categorized into SVA, PEER, HCP and PCA. Using 362 synthetic data sets and 110 real data sets, they found that not only does the PCA method underlies the statistical methodology behind the popular methods but is also orders of magnitude faster, better-performing, and much easier to interpret and use. The authors also provided an R package, PCAForQTL, along with a detailed users guide. The package is freely available at https://github.com/heatherjzhou/PCAForQTL. This work is interesting and fundamentally important in eQTL mapping. Hidden variable inference is the preliminary step in eQTL mapping. The PCA method for hidden variable inference is faster, better-performing and easier interpretation. This discovery is somewhat surprising. However, high efficiency of PCA relative to partial least squares for genomic prediction has been reported (Du et al. 2018), which agrees with the observation of this study. I fully support this study and recommend the paper to be published in Genome Biology after some minor revision. I have the following comments, which need to be considered in the revision.

1 The title does not appear to reflect the content of the article. I suggest to replace QTL mapping by eQTL mapping. Hidden variable inference for multiple molecular traits only applies to eQTL mapping, not to the conventional QTL mapping where agronomic and clinic traits are the response variables and genetic variants (genotypes) are independent variables.

2 Conventional QTL mapping emphasizes population structures inferred from genotype data. This study does not even discuss QTL mapping with population structures and cryptic covariance between individuals. The Matrix eQTL package only considers population structures but not hidden variables from molecular traits. I suggest that the authors discuss the possibility of modifying PCAForQTL to cover population structures and cryptic correlation between individuals.

3 It will be helpful to add a small numerical example for the PCA of molecular traits and the model for eQTL mapping. With this small numerical example, the paper will be easily understood by a general reader.

References

Du, C., Wei, J., Wang, S., Jia, Z., 2018. Genomic selection using principal component regression. Heredity 121, 12-23 (2018). https://doi.org/10.1038/s41437-018-0078-x

**Reviewer 2**

**Were you able to assess all statistics in the manuscript, including the appropriateness of statistical tests used?**

Yes

**Comments to author:**

DISCLAIMER: We have recently published results that are highly consistent with the observations presented in this manuscript (though on different methods and using different data, see Malik & Michoel, ref [46] in the manuscript). My review is based (but hopefully not biased) partly on that work.

In this manuscript, the authors present a thorough comparison between PCA and a number of methods for hidden variable inference in the context of eQTL mapping, most notably the popular PEER method that has been used extensively in the GTEx and other high-profile projects. Using simulated data, the authors show that PCA performs better in terms of recapturing hidden factors and bona fide eQTLs, while being orders of magnitude faster. In real data from the GTEx project, they show that PEER factors are almost identical to PCs and may be highly correlated to one another.

These results may come as a surprise to some, given the nearly uncontestable status that PEER has achieved within the eQTL data analysis community, but sadly they reflect the fact that computational biology methods can rise to fame almost by accident rather than by sound statistical arguments. It is telling that it has taken more than a decade since PEER was published for an analysis such as the present one to appear.

I strongly support publication of this manuscript and have some comments that I hope would further improve the presentation and interpretation of the results.

My major comment concerns the take-home messages of the manuscript. In simplified form, the authors state that (a) "PCA performs better than PEER and other methods" and (b) "PEER and other methods are related to PCA and their inferred factors overlap with PCs". It seems to me that these two statements are contradictory: if the PEER factors overlap with PCs, how can downstream performance be inferior to PCA? There are a number of points the authors could look into to clarify this apparent contradiction:

- It seems statement (a) is based mostly on the simulations, whereas statement (b) comes mainly from the analysis of the GTEx data. Can the authors perform some of the analyses they did on the simulated data also on the real data, and vice versa? For instance, correlation of PEER factors with each other and PCs is not done in the simulated data. Pseudo ground-truth eQTLs for AUPRC in the real data could be defined as eQTLs from an external source and then testing how many are found in GTEx by different methods and parameter settings, etc. If factors inferred by PEER and other methods are not independent, could some of the simulation results be explained by PEER et al. using a too low effective number of covariates compared to PCA?

- While PCs can be found exactly using the SVD, the other methods involve numerical optimization of an intractible cost function. That means failure of the other methods could be due to either the true optimal solution being inferior to PCA, or the numerical optimization not finding the true optimal solution, and therefore a suboptimal solution being used in the downstream analyses. In other words is the model "wrong" or is the optimization algorithm wrong? In our analysis of PANAMA, we were able to create data where the true solution to the model was known, and found that in fact PANAMA's optimization algorithm often doesn't converge to it. But when it did converge, the solution overlapped with PCA. Is a similar scenario at play in some of the analyses presented here? In general, I feel the authors arguments that PCA, SVA, PEER, HCP are closely related statistical methods are somewhat hand-waving and would benefit from a deeper analysis of the other methods' models and optimization algorithms.

A few minor comments:

- In section 2.1, for the benefit of the reader, say with respect to what the AUPRC are being measured (eQTL discovery). This is not clear without consulting the methods first.

- Figure 1a and Figure 6 are rather qualitative and somewhat subjective. I'm not sure if they merit being part of the main manuscript?

- In Figure 2, why are there no panels for the concordance scores? Most of what is in Fig S1 is equally informative as Fig 2, and the two analyses (true factor recovery and true eQTL recovery) are quite distinct in principle.

- In Figure 3, use of the relative AUPRC can be a bit confusing. I understand why the authors use it, but it may lead to the impression that the performance of the "unadjusted" method increases with the number of covariates, which of course does not make sense.

- I believe the method "PCA_resid_*" will do the same as our Lvreml method (ref [46]) and if correct, this could be mentioned.

- The authors briefly touch upon the problem of interpreting inferred hidden factors in the Discussion. They may wish to expand upon this in the context of their advice not to residualize w.r.t. known covariates. The authors are correct that residualizing is not needed for the analyses presented here, but it can actually be important when interpreting hidden factors, to make sure that inferred factors don't overlap with those known factors.

- The authors say that PANAMA is deprecated. I don't quite agree. PANAMA was published after PEER (by the same authors), and was shown to perform better than PEER in that publication. It is based on a more conventional and better understood linear mixed model that the same authors have later also applied on the analysis of single-cell data. I will not go as far as suggesting that the authors include PANAMA in their comparisons, because it would score a dark red in their speed and usability criteria, and as shown in our paper, the true solution of the PANAMA model (though not always found by their software) is essentially equivalent to the PCA_resid method already included in the analysis.

Tom Michoel

**Authors’ response**

**Point-by-point responses to the reviewers’ comments:**

**Reviewer #1:**

The authors compared 15 methods of hidden variable inference for eQTL mapping. These methods are categorized into SVA, PEER, HCP and PCA. Using 362 synthetic data sets and 110 real data sets, they found that not only does the PCA method underlies the statistical methodology behind the popular methods but is also orders of magnitude faster, better-performing, and much easier to interpret and use. The authors also provided an R package, PCAForQTL, along with a detailed users guide. The package is freely available at <https://github.com/heatherjzhou/PCAForQTL>. This work is interesting and fundamentally important in eQTL mapping. Hidden variable inference is the preliminary step in eQTL mapping. The PCA method for hidden variable inference is faster, better-performing and easier interpretation. This discovery is somewhat surprising. However, high efficiency of PCA relative to partial least squares for genomic prediction has been reported (Du et al. 2018), which

agrees with the observation of this study. I fully support this study and recommend the paper to be published in Genome Biology after some minor revision. I have the following comments, which need to be considered in the revision.

*We thank the reviewer for appreciating the value of our work and mentioning Du et al. 2018. We have added this reference in the Discussion section of our revised manuscript.*

1 The title does not appear to reflect the content of the article. I suggest to replace QTL mapping by eQTL mapping. Hidden variable inference for multiple molecular traits only applies to eQTL mapping, not to the conventional QTL mapping where agronomic and clinic traits are the response variables and genetic variants (genotypes) are independent variables.

*We thank the reviewer for this suggestion. Indeed, while conventional QTL mapping typically considers a small number of macro traits as the response variables, molecular QTL (which our paper concerns) typically considers a large number of molecular traits as the response variables, and hidden variable inference is only applicable in the latter case.*

*We have updated the title, abstract, and introduction to clarify that our paper concerns molecular QTL mapping rather than conventional QTL mapping. The reason we say “molecular QTL” rather than “eQTL” as the reviewer suggested is because our study is applicable to different kinds of molecular QTL analyses, including eQTL, sQTL, and 3’aQTL, not just eQTL (see Introduction).*

2 Conventional QTL mapping emphasizes population structures inferred from genotype data. This study does not even discuss QTL mapping with population structures and cryptic covariance between individuals. The Matrix eQTL package only considers population structures but not hidden variables from molecular traits. I suggest that the authors discuss the possibility of modifying PCAForQTL to cover population structures and cryptic correlation between individuals.

*We thank the reviewer for this comment. In molecular QTL analysis, researchers typically account for population structures by including the top genotype PCs as covariates in the linear regressions (e.g., in Matrix eQTL and in FastQTL). These top genotype PCs are typically considered known covariates. On the other hand, inferred covariates from molecular traits, such as PCs or PEER factors, can also be included as covariates in the linear regressions (e.g., in Matrix eQTL and in FastQTL). In other words, genotype PCs and inferred covariates from molecular traits are jointly used as covariates in QTL analysis. We have clarified this in the added numerical example in Methods. We’d also like to point the reviewer to the second to last paragraph of Discussion, where we discuss the connection and distinction between inferred covariates from molecular traits and genotype PCs.*

*Since the package PCAForQTL mainly concerns how to use PCA to obtain the inferred covariates from molecular traits, we did not update the package to “cover population structures”.*

3 It will be helpful to add a small numerical example for the PCA of molecular traits and the model for eQTL mapping. With this small numerical example, the paper will be easily understood by a general reader.

*We thank the reviewer for this suggestion. We have added a subsection in Methods to give a numerical example (see screenshot below).*

**Reviewer #2:**

DISCLAIMER: We have recently published results that are highly consistent with the observations presented in this manuscript (though on different methods and using different data, see Malik & Michoel, ref [46] in the manuscript). My review is based (but hopefully not biased) partly on that work.

In this manuscript, the authors present a thorough comparison between PCA and a number of methods for hidden variable inference in the context of eQTL mapping, most notably the popular PEER method that has been used extensively in the GTEx and other high-profile projects. Using simulated data, the authors show that PCA performs better in terms of recapturing hidden factors and bona fide eQTLs, while being orders of magnitude faster. In real data from the GTEx project, they show that PEER factors are almost identical to PCs and may be highly correlated to one another.

These results may come as a surprise to some, given the nearly uncontestable status that PEER has achieved within the eQTL data analysis community, but sadly they reflect the fact that computational biology methods can rise to fame almost by accident rather than by sound statistical arguments. It is telling that it has taken more than a decade since PEER was published for an analysis such as the present one to appear.

I strongly support publication of this manuscript and have some comments that I hope would further improve the presentation and interpretation of the results.

*We thank Dr. Michoel for the strong support and insightful comments.*

My major comment concerns the take-home messages of the manuscript. In simplified form, the authors state that (a) "PCA performs better than PEER and other methods" and (b) "PEER and other methods are related to PCA and their inferred factors overlap with PCs". It seems to me that these two statements are contradictory: if the PEER factors overlap with PCs, how can downstream performance be inferior to PCA? There are a number of points the authors could look into to clarify this apparent contradiction:

- It seems statement (a) is based mostly on the simulations, whereas statement (b) comes mainly from the analysis of the GTEx data. Can the authors perform some of the analyses they did on the simulated data also on the real data, and vice versa? For instance, correlation of PEER factors with each other and PCs is not done in the simulated data. Pseudo ground-truth eQTLs for AUPRC in the real data could be defined as eQTLs from an external source and then testing how many are found in GTEx by different methods and parameter settings, etc. If factors inferred by PEER and other methods are not independent, could some of the simulation results be explained by PEER et al. using a too low effective number of covariates compared to PCA?

- While PCs can be found exactly using the SVD, the other methods involve numerical optimization of an intractible cost function. That means failure of the other methods could be due to either the true optimal solution being inferior to PCA, or the numerical optimization not finding the true optimal solution, and therefore a suboptimal solution being used in the downstream analyses. In other words is the model "wrong" or is the optimization algorithm wrong? In our analysis of PANAMA, we were able to create data where the true solution to the model was known, and found that in fact PANAMA's optimization algorithm often doesn't converge to it. But when it did converge, the solution overlapped with PCA. Is a similar scenario at play in some of the analyses presented here? In general, I feel the authors arguments that PCA, SVA, PEER, HCP are closely related statistical methods are somewhat hand-waving and would benefit from a deeper analysis of the other methods' models and optimization algorithms.

*We thank Dr. Michoel for these insightful comments. We have expanded Section 2.3 and added Figure S8 (see screenshots below) to reconcile these two seemingly contradictory facts: (1) PCA outperforms PEER in our simulation studies, and (2) PEER factors and PCs are highly similar in GTEx eQTL and sQTL data. The intuition of our conclusion is that although PEER factors and PCs can be highly similar, as long as they are not exactly identical, there can still be some performance differences (and PCA is the better performer).*

*Regarding numerical optimization, PEER is based on a Bayesian model. The inference is performed using variational Bayes and in particular, initialized with PCs. Therefore, the initialization shouldn’t be why PEER factors diverge from PCs. In fact, as we see in Figure S8, PEER factors can be quite similar to PCs. SVA is purely algorithmic and is not defined based on a model or objective function, so optimization is not needed. HCP is defined by minimizing a loss function. The loss function is simple enough that it is differentiable, so coordinate descent is used. We have expanded Section 2.4 to include these details. In the updated Section 2.4, we also point the readers to Zhou [41] more explicitly, where we have previously reviewed PCA, SVA, PEER, and HCP in detail, including their optimization algorithms (when applicable).*

A few minor comments:

- In section 2.1, for the benefit of the reader, say with respect to what the AUPRC are being measured (eQTL discovery). This is not clear without consulting the methods first.

*We thank Dr. Michoel for this suggestion. We have updated the text to specify that it is the AUPRC of the QTL result.*

- Figure 1a and Figure 6 are rather qualitative and somewhat subjective. I'm not sure if they merit being part of the main manuscript?

*We thank Dr. Michoel for this comment. For Figure 1a, we have been careful to justify the rankings of the different methods in the caption. We feel that Figure 1a is worth keeping in the main text because it gives an overview of our results. The reason we rate SVA and PEER as less interpretable than PCA and HCP is because we believe that methods that can be defined by simple loss functions are more interpretable than (1) algorithms involving complicated steps with no underlying model or loss function (SVA) and (2) methods based on multilayered Bayesian models that require variational Bayes to approximate (PEER). We hope the Dr. Michoel agrees.*

*We have slightly modified Figure 6 to make it less potentially subjective (changed “is heavily based on” to “iteratively performs”). We feel that Figure 6 is worth keeping in the main text as well because it gives an overview of the connections between the different methods from a theoretical perspective. The statistical statements in Figure 6 and Section 2.4 are justified in either this paper or Zhou [41].*

- In Figure 2, why are there no panels for the concordance scores? Most of what is in Fig S1 is equally informative as Fig 2, and the two analyses (true factor recovery and true eQTL recovery) are quite distinct in principle.

*We thank Dr. Michoel for this question. We considered both options: including the concordance score comparisons in Figure 2 (see version below) and not including them (see version in the manuscript). Both versions of the plot could make sense, but we chose the version without the concordance score comparisons for a few reasons:*

*1.	Brevity. Even if we do include the concordance score comparisons in Figure 2, we would still want to keep Figure S1(e) in Figure S1 because the three adjusted R^2^ measures (adjusted R^2^, reverse adjusted R^2^, and concordance score) are best understood together, since concordance score is defined as the average of the other two measures. Further, if we do include the concordance score comparisons in Figure 2, for consistency, we would also want to include another row of subplots in Figure 3 for concordance score (but still keep Figure S4(c) in Figure S4). In short, Figure S1(e) and Figure S4(c) would each be shown twice, once in the main text and once in the supplement.*

*2.	Focus. We think of AUPRC as the main performance evaluation criterion and concordance score as a supporting criterion because hidden variable inference is a means to an end—namely QTL detection. This is why in Section 2.1, we frame the observation that PCA has the highest average concordance scores as an explanation of why PCA has the highest AUPRCs rather than a “victory” in its own right.*

*3.	Incomplete agreement between AUPRC and concordance score. This speaks to our previous point. While a method having a higher average concordance score usually means it has a higher average AUPRC, as we can see from the plot below, this is not always the case. For example, consider*

*a.	In Simulation Design 1: Method 9 vs. Method 11; Method 9 vs. Method 15.*

*b.	In Simulation Design 2: Method 5 vs. Method 9; Method 9 vs. Method 15.*

*4.	Visual similarity between Figure 2 and Figure S1. This is not a major concern, but if we adopt the plot below for Figure 2, Figure 2 would look very similar to Figure S1 (both are 3 by 2 grids with the same legend). We think it’s beneficial to keep the plots more visually distinct.*

*5.	Confusion that some bars are identical. In (e) and (f) below, whether the factor approach or the residual approach is used in PEER does not affect how the bar looks. For example, the bars for Method 7 and Method 8 are identical to each other in both (e) and (f). While this is not a problem, it may lead to unnecessary confusion for some readers.*

*Again, we thank Dr. Michoel for this question. After deliberation, we chose to keep figures related to concordance scores in the supplement.*

- In Figure 3, use of the relative AUPRC can be a bit confusing. I understand why the authors use it, but it may lead to the impression that the performance of the "unadjusted" method increases with the number of covariates, which of course does not make sense.

*We thank Dr. Michoel for this comment. We have done two things to make this clearer. First, in Figure 2(d) and Figure 3(b), we have changed the y-axis labels from decimals to percentages so that in Figure 3(b), -3% (rather than -0.03), for example, can be more easily understood as “the AUPRC of a certain method is 3% lower than that of Ideal”.*

*The second thing is that we have updated the caption of Figure 3 to clarify that numOfCovariates refers to the number of simulated covariates (as opposed to the number of inferred covariates).*

*Hopefully these changes will make it clearer that it’s not that the performance of Unadjusted increases with the number of inferred covariates, but that the performance gap between Unadjusted and Ideal decreases with the number of simulated covariates.*

- I believe the method "PCA_resid_*" will do the same as our Lvreml method (ref [46]) and if correct, this could be mentioned.

*We thank Dr. Michoel for this comment. We tried LVREML on the first simulated data set of Simulation Design 1 and found that the concordance between the inferred covariates produced by LVREML (setting K=7, the true number of hidden covariates) and the top seven PCs produced by PCA_resid is 0.84. Here, the “concordance” is defined analogously as the concordance score in the manuscript. Specifically, the adjusted R^2^’s from predicting each PC using all seven LVREML factors are 0.9964213, 0.9935429, 0.9984576, 0.9819956, 0.9941219, 0.7027608, and 0.2220948, and the adjusted R^2^’s from predicting each LVREML factor using all seven PCs are 0.9945417, 0.9655849, 0.8456639, 0.9929742, 0.9480600, 0.8362354, and 0.8792585. In other words, we found that LVREML is not equivalent to PCA_resid.*

- The authors briefly touch upon the problem of interpreting inferred hidden factors in the Discussion. They may wish to expand upon this in the context of their advice not to residualize w.r.t. known covariates. The authors are correct that residualizing is not needed for the analyses presented here, but it can actually be important when interpreting hidden factors, to make sure that inferred factors don't overlap with those known factors.

*We thank Dr. Michoel for this comment. We agree that residualization ensures low correlation or near independence between the known covariates and the PCs. However, we are not sure why this helps with the interpretation of the PCs because usually inferred covariates are considered explained or interpreted when some known covariates can be found that are strongly correlated with the inferred covariates. For example,* [*https://doi.org/10.1371/journal.pcbi.1009939*](https://doi.org/10.1371/journal.pcbi.1009939) *states, “This pipeline increases the sensitivity of eQTL mapping, but has remained challenging to interpret because PEER factors a priori are not strongly associated with any known biological or technical source”.*

- The authors say that PANAMA is deprecated. I don't quite agree. PANAMA was published after PEER (by the same authors), and was shown to perform better than PEER in that publication. It is based on a more conventional and better understood linear mixed model that the same authors have later also applied on the analysis of single-cell data. I will not go as far as suggesting that the authors include PANAMA in their comparisons, because it would score a dark red in their speed and usability criteria, and as shown in our paper, the true solution of the PANAMA model (though not always found by their software) is essentially equivalent to the PCA_resid method already included in the analysis.

*We thank Dr. Michoel for this comment. We have removed the word “deprecated” from our manuscript. We previously said that PANAMA was deprecated because the link provided in the LVREML paper for the PANAMA package,* [*https://github.com/limix/limix-legacy*](https://github.com/limix/limix-legacy)*, said that the package was deprecated.*

**Second round of review**

**Reviewer 1**

The authors have adequately addressed all my concerns. This manuscript is now acceptable.

**Reviewer 2**

The authors have carefully addressed all my previous comments and I have no additional comments.
